# Belatacept Does Not Inhibit Follicular T Cell-Dependent B-Cell Differentiation in Kidney Transplantation

**DOI:** 10.3389/fimmu.2017.00641

**Published:** 2017-05-31

**Authors:** Gretchen N. de Graav, Dennis A. Hesselink, Marjolein Dieterich, Rens Kraaijeveld, Wenda Verschoor, Dave L. Roelen, Nicolle H. R. Litjens, Anita S. Chong, Willem Weimar, Carla C. Baan

**Affiliations:** ^1^Department of Internal Medicine, Section Transplantation and Nephrology, Erasmus MC, University Medical Center, Rotterdam, Netherlands; ^2^Department of Immunohematology and Blood Transfusion, Leiden University Medical Center, Leiden, Netherlands; ^3^Department of Surgery, The University of Chicago, Chicago, IL, United States

**Keywords:** belatacept, costimulatory blockade, follicular T-helper cells, immunoglobulins, plasmablasts, tacrolimus, transitional B-cells

## Abstract

Humoral alloreactivity has been recognized as a common cause of kidney transplant dysfunction. B-cell activation, differentiation, and antibody production are dependent on IL-21^+^CXCR5^+^follicular T-helper (Tfh) cells. Here, we studied whether belatacept, an inhibitor of the costimulatory CD28-CD80/86-pathway, interrupts the crosstalk between Tfh- and B-cells more efficiently than the calcineurin inhibitor tacrolimus. The suppressive effects of belatacept and tacrolimus on donor antigen-driven Tfh–B-cell interaction were functionally studied in peripheral blood mononuclear cells from 40 kidney transplant patients randomized to a belatacept- or tacrolimus-based immunosuppressive regimen. No significant differences in uncultured cells or donor antigen-stimulated cells were found between belatacept- and tacrolimus-treated patients in the CXCR5^+^Tfh cell generation and activation (upregulation of PD-1). Belatacept and tacrolimus *in vitro* minimally inhibited Tfh-cell generation (by ~6–7%) and partially prevented Tfh-cell activation (by ~30–50%). The proportion of IL-21^+^-activated Tfh-cells was partially decreased by *in vitro* addition of belatacept or tacrolimus (by ~60%). Baseline expressions and proportions of activated CD86^+^ B-cells, plasmablasts, and transitional B-cells after donor antigen stimulation did not differ between belatacept- and tacrolimus-treated patients. Donor antigen-driven CD86 upregulation on memory B-cells was not fully prevented by adding belatacept *in vitro* (~35%), even in supratherapeutic doses. In contrast to tacrolimus, belatacept failed to inhibit donor antigen-driven plasmablast formation (~50% inhibition vs. no inhibition, respectively, *p* < 0.0001). In summary, donor antigen-driven Tfh-B-cell crosstalk is similar in cells obtained from belatacept- and tacrolimus-treated patients. Belatacept is, however, less potent *in vitro* than tacrolimus in inhibiting Tfh-cell-dependent plasmablast formation.

## Introduction

B-cells and antibodies against the allograft are increasingly recognized to contribute to alloreactivity and subsequent graft failure after kidney transplantation under the currently used calcineurin inhibitor (CNI)-based immunosuppressive regimen (1–7). CD4^+^CXCR5^+^follicular T-helper (Tfh) cells are key mediators in B-cell activation, differentiation, and antibody production (8–12). Moreover, these cells infiltrate the allograft and colocalize with B-cells during acute rejection after kidney transplantation (13, 14). In alloreactivity, both Tfh- and B-cells are activated by the same antigen *via* their T- and B-cell receptor, respectively (15). The CD40-40L, CD28-CD80/86, and ICOS-ICOSL costimulatory pathways and the cytokines IL-6 and IL-21 are important in this Tfh–B-cell interaction and for B-cell differentiation into immunoglobulin-producing plasma cells (16–21).

Belatacept is a selective inhibitor of the CD28-CD80/86 pathway and subsequently interrupts Tfh–B-cell interaction (21, 22). In animal transplant models, belatacept, or the lower affinity version abatacept (CTLA4 Immunoglobulin), inhibited germinal center formation, clonal B-cell expansion, IL-21 production, and the development of donor-specific anti-human leukocyte antigen antibodies (DSA) (14, 23). These findings were in line with observations from a large randomized, controlled trial in kidney transplant patients where the belatacept-based regimen resulted in a significantly lower prevalence of DSA than the cyclosporine A (CsA)-based regimen at 7 years after transplantation: 4.6 vs. 17.8%, respectively (24). However, in all these clinical studies, belatacept was combined with other immunosuppressive drugs: in the BENEFIT and BENEFIT-EXT trials belatacept was combined with mycophenolate mofetil (MMF) and prednisone, and in the animal studies, belatacept was combined with either sirolimus or T-cell-depleting antibodies (14, 23–25).

Contradictory effects of tacrolimus on B-cell activation, proliferation, and differentiation have been reported (26–28) because tacrolimus only inhibits calcium-influx dependent and not calcium-independent, B- and T-cell activation (27, 29). This calcineurin-mediated activation is dependent on the type of stimulus (26, 28, 29). B-cell activation can thus be prevented by calcineurin-inhibition in an antigen-dependent manner. The effect of tacrolimus on donor antigen-stimulated Tfh–B-cell interaction is unknown in kidney transplantation.

In addition to the *in vivo* animal studies and clinical data that suggest belatacept effectively inhibits the humoral immune response specific for donor antigen (14, 23, 24), this class of immunosuppressive agents may also favor a more regulatory rather than effector alloreactive B-cell activity by enhancing the survival of transitional B-cells over memory B-cells in the long term (30). Theoretically, this may reduce rejection risk (15, 30–34).

So far no studies have been conducted which compared the effects of belatacept to tacrolimus, on Tfh–B-cell interaction in kidney transplantation. We hypothesized that belatacept more efficiently interrupts Tfh-B-cell crosstalk than tacrolimus. Therefore, we compared (i) the frequencies of Tfh and B-cell subsets between belatacept- and tacrolimus-treated patients; (ii) the *in vitro* donor antigen-driven Tfh–B-cell interaction in peripheral blood mononuclear cells (PBMCs) obtained from belatacept- and tacrolimus-treated kidney transplant patients; and (iii) the isolated the effects of *additional* belatacept and tacrolimus *in vitro* on donor antigen-driven Tfh–B-cell interaction in PBMCs obtained from the same patients.

## Materials and Methods

### Study Population and Materials

Materials were collected from 40 kidney transplant patients and their donors who participated in a prospective, randomized-controlled trial (approved by the Medical Ethical Committee of the Erasmus MC, University Medical Centre Rotterdam; MEC-2012-42, EUDRACT CT # 2012-003169-16). After written informed consent, patients were included and randomized to a tacrolimus-based (control) or belatacept-based (experimental) immunosuppressive regimen. For in- and exclusion criteria, refer to Table S1 in Supplementary Material. All procedures were in accordance with the ethical standards of the Declaration of Istanbul (35). In short, both groups received basiliximab induction therapy (Simulect^®^, Novartis, Basel, Switzerland), followed by maintenance therapy with MMF and prednisolone, which was tapered to 5 mg by month 3 after transplantation. Maintenance therapy with tacrolimus (Prograf^®^, Astellas Pharma, Tokyo, Japan) was adjusted to predose levels of 5–10 ng/mL, while belatacept (Nulojix^®^, Bristol-Meyers Squibb, NYC, NY, USA) was dosed according to bodyweight (Less-Intensive regimen of the BENEFIT trials) (36).

Lithium heparin blood was collected from patients 1 day before transplantation and 3 months after transplantation or during clinically suspected acute rejection before any additional anti-rejection therapy was given. All samples were processed within 24 h of withdrawal. If patients had a biopsy-proven acute rejection (BPAR) (2) materials of that time point were used instead of their materials of 3 months after transplantation. Lithium heparinized blood from donors was collected 1 day before transplantation. PBMCs were isolated from blood using the Ficoll density isolation method.

### Mixed Lymphocyte Reactions (MLRs)

Patients PBMCs obtained after transplantation were thawed and used in MLRs. PBMCs were obtained 3 months after transplantation in stable, non-rejecting patients or before additional antirejection therapy was given in rejecting patients. Live cells were counted under a light microscope and distinguished from dead cells with Trypan Blue. Per patient ~5 × 10^5^ uncultured PBMCs were stained for phenotypical analyses. A total of 5 × 10^4^ patients’ PBMCs/well (in a 96-wells plate) were stimulated for 7 days at 37°C with 5 × 10^4^ carboxyfluorescein succinimidyl ester (CFSE)-labeled, irradiated donor PBMCs (40 Gy) in RPMI 1640 + 10% heat-inactivated fetal bovine serum. Half of patients’ PBMCs were incubated for 1 h with clinically therapeutic concentrations of belatacept (10 μg/mL) (37) or tacrolimus (10 ng/mL), dependent on the randomization group, before donor antigen was added. After the donor antigen was added, these amounts of immunosuppressive drugs remained in the culture for the whole period of 7 days. At the end of day 6, 100 µL supernatant per well was harvested and stored at −20°C. Subsequently, Monensin and Brefeldin (GolgiStop and GolgiPlug, BD Biosciences, Franklin Lakes, NJ, USA) were added for 16 h over night in a concentration of 1:1,500 and 1:1,000, respectively, to allow the measurement of intracellularly accumulated cytokines in PBMCs.

**Figure 1:**
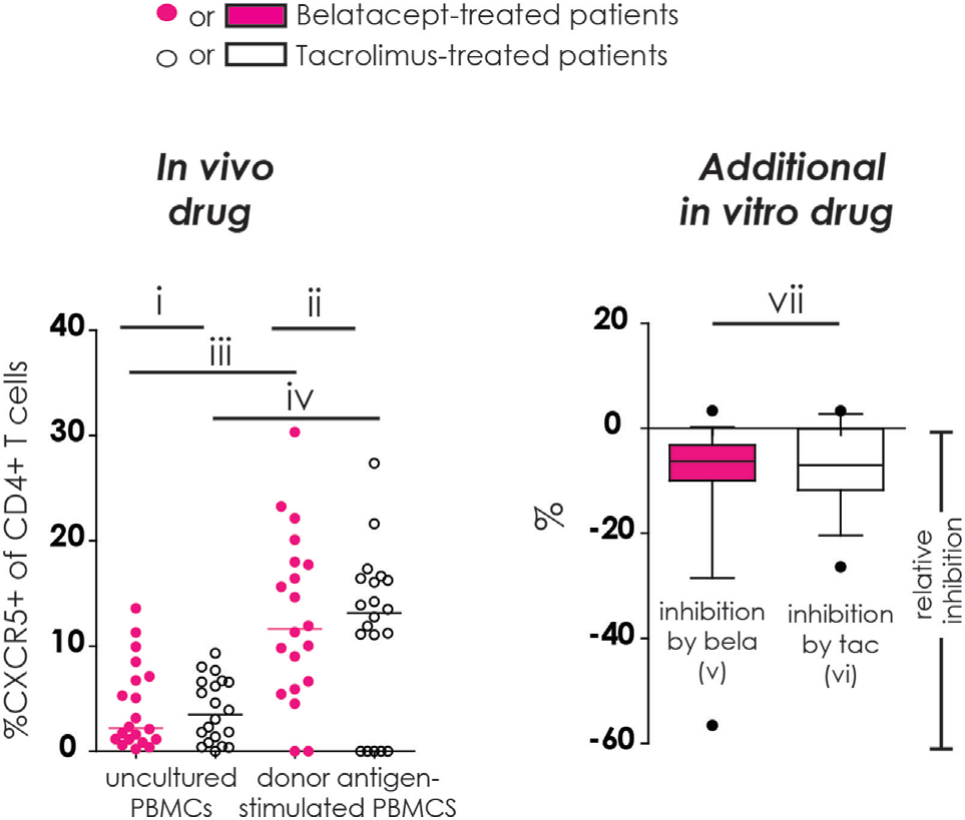
Different comparisons made in conducted studies (example figure). All figures in this manuscript comprise seven different comparisons. In this example, the proportion of CXCR5^+^ cells within CD4^+^ T-cells was used. In the left column (“*in vivo* drug”), the proportions of studied cell populations (see Flow Cytometry and Materials and Methods) were compared between (i) belatacept- and tacrolimus-treated patients in uncultured, unstimulated peripheral blood mononuclear cells (PBMCs); (ii) belatacept- and tacrolimus-treated patients in 7-day donor antigen-stimulated PBMCs; (iii) uncultured, unstimulated PBMCs and 7-day donor antigen-stimulated PBMCs in belatacept-treated patients; and (iv) uncultured, unstimulated PBMCs and 7-day donor antigen-stimulated PBMCs in tacrolimus-treated patients. In the right column (“Additional *in vitro* drug”), the relative inhibition by additional *in vitro* belatacept or tacrolimus is depicted by “v” and “vi”, respectively, in this example figure. If the median relative inhibition is significantly smaller than 0, the *in vitro* drug significantly decreases the proportion of the studied cell type. Per cell type, the relative inhibitions were compared between belatacept and tacrolimus *in vitro*. This is depicted by “vii” in this example figure. Refer to Figure 2B for the population used in this example (proportion of CXCR5^+^ within CD4^+^ T cells).

Refer to Figure 1 for the different comparisons made in our study. Proportions of studied cell populations (see Flow Cytometry) were compared between:

(i) belatacept- and tacrolimus-treated patients in uncultured, unstimulated PBMCs;
(ii) belatacept- and tacrolimus-treated patients in 7-day donor antigen-stimulated PBMCs;
(iii) uncultured, unstimulated PBMCs and 7-day donor antigen-stimulated PBMCs in belatacept-treated patients;
(iv) uncultured, unstimulated PBMCs and 7-day donor antigen-stimulated PBMCs in tacrolimus-treated patients.

Patient PBMCs obtained 1 day before transplantation were also cultured with donor antigen in the same way to investigate whether PBMCs obtained from an immunosuppressed environment reacted differently on donor antigen compared to PBMCs before any immunosuppression was given.

Post-transplant PBMCs obtained from three belatacept-treated and three tacrolimus-treated patients were used in MLRs to study the CD40-CD40L, PD1-PDL1, and ICOS-ICOSL interaction during costimulation blockade by belatacept 10 µg/mL or calcineurin-inhibition by tacrolimus 10 ng/mL. MLRs were conducted as described above, only with 5 × 10^4^ CD3 and CD19-depleted irradiated donor PBMCs instead of CFSE-labeled donor PBMCs. The same methods were used in six independent MLRs of healthy controls’ PBMCs to determine free CD80/86 expression after allo-antigen stimulation in the presence of various concentrations of belatacept (0–1,000 µg/mL) or tacrolimus (0–100 ng/mL).

### CFSE Labeling of PBMCs

To distinguish between patient and donor PBMCs in the MLRs, donor PBMCs were labeled with the cell-permeable, intracellular linker CFSE (Thermo Fisher Scientific, Waltham, MA, USA), according to manufacturer’s manual. CFSE-labeled donors’ PBMCs expressed as median fluorescence intensity (MFI) >10^4^ on the FITC channel.

### Cocultures of Isolated Follicular T-Helper (Tfh) Cells and Memory B-Cells (13, 38)

CD3^+^CD4^+^CXCR5^+^ T-cells and CD19^+^CD27^+^ B-cells from three healthy controls and three patients before transplantation were isolated using a FACSAria II 4L SORP™ (BD Biosciences). From both populations 2 × 10^4^ cells/well were cocultured for 7 days at 37°C with 4 × 10^4^ 40 Gy irradiated CD3/CD19-depleted, allogeneic PBMCs. Half of the wells were spiked with belatacept 10 µg/mL or tacrolimus 10 ng/mL. After 7 days coculture, supernatants were collected and stored at −20°C until analysis, and the proportion of memory B-cells that differentiated into CD27^+^CD38^++^ plasmablasts was measured.

### Flow Cytometry

For a complete overview of the monoclonal antibodies used, see Table S2 in Supplementary Material. Follicular T-helper (Tfh) cells were defined as CD3^+^CD4^+^CXCR5^+^ T lymphocytes and classified as activated or resting by their expression of the activation marker and coinhibitor PD-1 (39, 40). Tfh-cell generation was defined as an increase in the proportion of CXCR5^+^ within CD4^+^ T-cells; Tfh-cell activation comprised the increase in the proportion of PD-1^+^ within CD4^+^CXCR5^+^ T-cells; and the generation of activated Tfh-cells was equivalent to an increase in the proportion of CXCR5^+^PD-1^+^ within CD4^+^ T-cells. The characteristic Tfh-cell cytokine IL-21 was determined in donor antigen-stimulated PBMCs in the presence or absence of belatacept or tacrolimus.

Within CD19^+^ B-cells, we distinguished CD27^−^ naïve B-cells, CD27^+^ memory B-cells, CD24^+^CD38^++^ transitional B-cells, and CD27^+^CD38^++^ plasmablasts. Free CD86 expression on B-cells was measured on donor antigen-stimulated PBMCs by using an antibody that is competitive with belatacept for CD86 but binds with lower affinity (41). Expressions of the immune regulatory cytokine IL-10 in transitional B-cells and of the aggressive effector cytokine TNFα in plasmablasts were also assessed after 7 days of donor antigen stimulation.

### ELISA for IgM and IgG3 Measurements

IgM concentrations in supernatants from all cell cultures were determined by ELISA. A calibration curve using human IgM 1.6–100 ng/mL (Sigma-Aldrich, St. Louis, MO, USA) was used to quantify results. All experiments were performed *in duplo* (medians were used for end result). Supernatants were diluted, if necessary, to fit within the measurements of the calibration curve. Measurements <1.6 ng/mL were considered negative. IgG3 concentrations were measured in the same way using an ELISA-kit with a calibration curve of 4.4–200 ng/mL (Affymetrix/eBioscience, Santa Clara, CA, USA).

### Single Bead Luminex Assay

DSA were measured in (14–150×) concentrated culture supernatants using the Single Antigen beads mix from the LABScreen Single Antigen class II kit (Thermo Fisher, Waltham, MA, USA) (13). Microbeads were analyzed with a Luminex LabscanTM 100 analyzer using the Luminex 100IS software and analyzed using the HLA Fusion 3.0 software. All samples fulfilled the quality criteria for reactivity of the control beads.

### Calculation of the Relative Inhibition

The relative inhibition was used to account for inter-patient variability in the response to donor antigen (Figure 1, “*additional in vitro*” column). The relative inhibition by *additional in vitro* belatacept or tacrolimus was calculated for the donor antigen-driven Tfh-cell generation, Tfh-cell activation, and the generation of activated Tfh-cells as well as for the donor antigen-driven intracellular IL-21 by activated Tfh-cells and the formation of IL21^+^ activated Tfh-cells. The relative inhibition by the *in vitro* drugs was also assessed for the upregulation of CD86 on naïve and memory B-cells, the formation of plasmablasts and their IgM production, and the transitional B-cell survival. For these calculations, the proportions of aforementioned cell subsets after donor antigen stimulation were set to 0 by using the following equation:

Relative inhibition (after donor  antigen stimulation)=(“proportion in the presence of  in vitro drug”− “proportion without  in vitro drug added”)“proportion without  in vitro drugs added”×100%

If the median relative inhibition is significantly smaller than 0, the *in vitro* drug significantly decreases the proportion of the studied cell type (Figure 1, comparison “v” and “vi”). Per cell type, the relative inhibitions were compared between belatacept and tacrolimus *in vitro* (Figure 1, comparison “vii”).

### Statistical Analyses

Proportions of cell subsets in uncultured or donor antigen-stimulated PBMCs were compared between the belatacept and tacrolimus group using the Mann–Whitney *U* test (Figure 1, comparisons “i” and “ii”) as well as baseline characteristics that were continuous variables. Baseline characteristics that were categorical variables were compared with the Fisher’s exact test. Proportions of cell subsets between uncultured and donor antigen-stimulated PBMCs were compared using the Wilcoxon signed rank test (Figure 1, comparisons “iii” and “iv”). The median relative inhibition was compared to a theoretical mean of 0 (=no inhibition) with the Wilcoxon signed rank test to determine if the inhibition by the *in vitro* drug was statistically significant (Figure 1, comparisons “v” and “vi”). The relative inhibitions by *in vitro* belatacept and *in vitro* tacrolimus were compared using the Mann–Whitney *U* test (Figure 1, comparison “vii”).

Multivariable linear regressions were used to examine the *in vitro* effects of belatacept compared to tacrolimus on donor antigen-activated Tfh and B-cell subsets, adjusted for confounders [presence of *in vitro* added drugs (present vs. absent), time point (after vs. before transplantation), and BPAR (PBMCs obtained during rejection vs. 3 months after transplantation)]. To avoid multiple testing errors, only cell subsets in which the relative inhibition significantly differed between belatacept and tacrolimus *in vitro* were included for these analyses.

SPSS Statistics 21.0 (IBM, Armonk, NY, USA) was used for statistical analyses. Unless mentioned otherwise, medians (+range) are given for continuous variables, and numbers (+proportions) are given for categorical variables. *p-*Values with a two-sided α of <0.05 were considered statistically significant.

## Results

### Study Population

No significant differences were observed with regard to baseline characteristics between the two treatment groups (Table S3 in Supplementary Material).

### The Effects of Belatacept and Tacrolimus on Follicular T-Helper (Tfh) cells

#### Tfh-Cell Generation and Activation (CXCR5 and PD-1 Upregulation)

**Figure 2:**
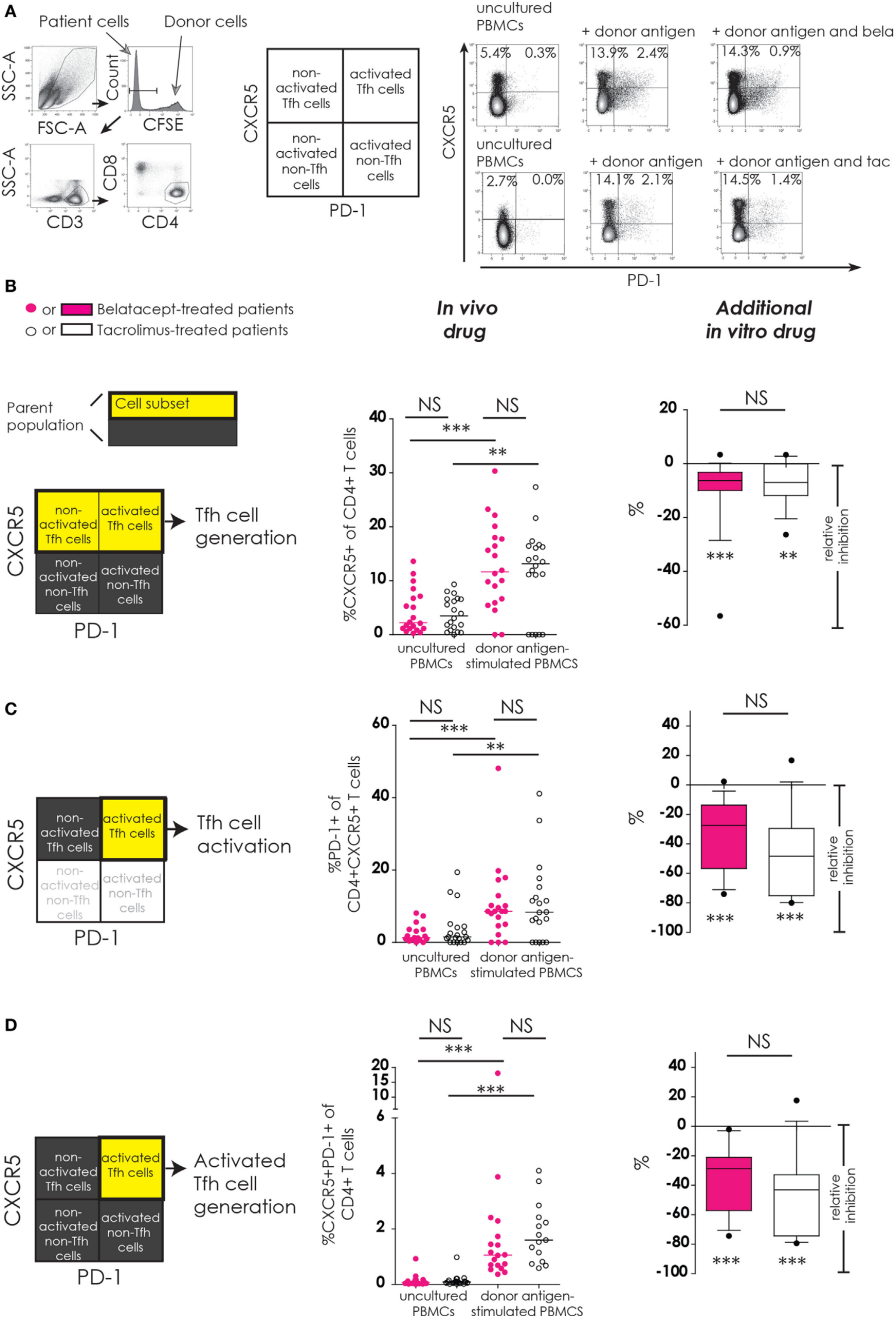
No differences between belatacept and tacrolimus *in vivo* or *in vitro* on donor antigen-driven follicular T-helper (Tfh) cell formation and activation in cultured peripheral blood mononuclear cells (PBMCs). Two typical examples are depicted for CXCR5 and PD-1 expression on Tfh cells in uncultured PBMCs and after 7 days of donor antigen stimulation, in the presence or absence of belatacept and tacrolimus **(A)**. Follicular T-helper (Tfh) cells were distinguished from non-Tfh-cells by surface CXCR5 expression, while activated cells were defined by surface PD-1 expression. Donor PBMCs were discriminated by carboxyfluorescein succinimidyl ester labeling them prior to the mixed lymphocyte reaction and gating them out after. The proportions are depicted of CXCR5^+^ within CD4^+^ T-cells **(B)**; PD-1^+^ within CD4^+^CXCR5^+^ T-cells **(C)**; and CXCR5^+^PD-1^+^ within CD4^+^ T-cells **(D)**. In the graphs in the “*In vivo drug*” column, proportions of aforementioned cell populations were compared (i) between belatacept- and tacrolimus-treated patients in uncultured, unstimulated PBMCs; (ii) between belatacept- and tacrolimus-treated patients in 7-day donor antigen stimulated PBMCs; (iii) between uncultured, unstimulated PBMCs and 7-day donor antigen stimulated PBMCs in belatacept-treated patients; and (iv) between uncultured, unstimulated PBMCs and 7-day donor antigen stimulated PBMCs in tacrolimus-treated patients. Every dot represents PBMCs of a single patient. In the graphs in the “*additional in vitro drug*” column the relative inhibitions by *additional in vitro* belatacept and tacrolimus are depicted for aforementioned cell populations in the same belatacept- and tacrolimus-treated patients. The proportions of these cell populations after donor antigen stimulation in the absence of *in vitro* drugs are set to 0. The median relative inhibitions by belatacept and tacrolimus were tested against a theoretical median of 0. Asterisks below the boxes depict the *p*-values of these tests. The relative inhibitions were compared between belatacept and tacrolimus *in vitro*. Lines in boxes represent medians, borders of boxes represent 25th and 75th percentiles, and error bars present 10th and 90th percentiles. Every box represents cultures of PBMCs obtained from *n* = 20 belatacept-treated or *n* = 20 tacrolimus-treated patients. **p < 0.01, ***p < 0.001, NS, not significant.

Refer to Figure 1 (example figure using the data from Figure 2B) for the different comparisons made in our study for the different cell subsets (more details in Section “Materials and Methods”). Results start from Figure 2. The surface expression of the Tfh marker CXCR5 and the activation marker PD-1 were determined on CD4^+^ Tfh cells (Figure 2).

Baseline expression of CXCR5 on CD4^+^ T-cells in uncultured PBMCs was comparable between belatacept- and tacrolimus-treated patients (Figure 2B, “*in vivo drug”* column). Following donor antigen stimulation, Tfh-cell generation, defined by the expression of CXCR5 on CD4^+^ T-cells, increased~3- to 4-fold in PBMCs obtained from belatacept- and tacrolimus-treated patients, *p* < 0.001 and *p* < 0.01, respectively (Figure 2B, “*in vivo drug”* column). This process was inhibited when the samples were spiked *in vitro* by adding tacrolimus and belatacept. The relative inhibition of Tfh-cell generation, however, was similar between belatacept and tacrolimus: −6.3% (−56.6 to +3.3%), *p* < 0.001, by belatacept and −7.0% (−26.4 to +3.3%), *p* < 0.01, by tacrolimus (Figure 2B, “*additional in vitro drug*” column).

The expression of PD-1 on CD4^+^CXCR5^+^ T-cells in uncultured PBMCs was similarly low in belatacept- and tacrolimus-treated patients (medians 1.3 and 1.5%, respectively; Figure 2C, “*in vivo drug*” column). Tfh-cells of belatacept- and tacrolimus-treated patients were significantly activated after CD4^+^ T-cells donor antigen stimulation, i.e., a significant increase of PD-1 expression on CXCR5^+^ was observed. The relative inhibition of Tfh-cell activation was −27.5% (−74.0 to +2.3%), *p* < 0.001, by belatacept and −48.4% (−80.0 to 16.7%), *p* < 0.001, by tacrolimus, inhibition by belatacept vs. tacrolimus; *p* = 0.13 (Figure 2C, “*additional in vitro drug*” column).

The proportion of CXCR5^+^PD-1^+^ double-positive CD4^+^ T-cells was negligible in uncultured PBMCs from belatacept- and tacrolimus-treated patients (Figure 2D, “*in vivo drug*” column). The generation of activated Tfh-cells (defined by an increase of the proportion of CXCR5^+^PD-1^+^ double-positive CD4^+^ T-cells) was 1.1% (0.4–18.1%) in donor antigen-stimulated PBMCs from belatacept-treated patients and 1.6% (0.6–4.1%) in those from tacrolimus-treated patients. These proportions were not significantly different. The generation of activated Tfh-cells was inhibited by both belatacept and tacrolimus *in vitro* (Figure 2D, “*additional in vitro drug*” column): the relative inhibition was −28.8% (−74.3 to −2.1%), *p* < 0.001 by belatacept, and −32.9% (−79.4 to +17.5%) by tacrolimus, *p* < 0.001.

### Tfh Cell Function (Intracellular IL-21 Production)

**Figure 3:**
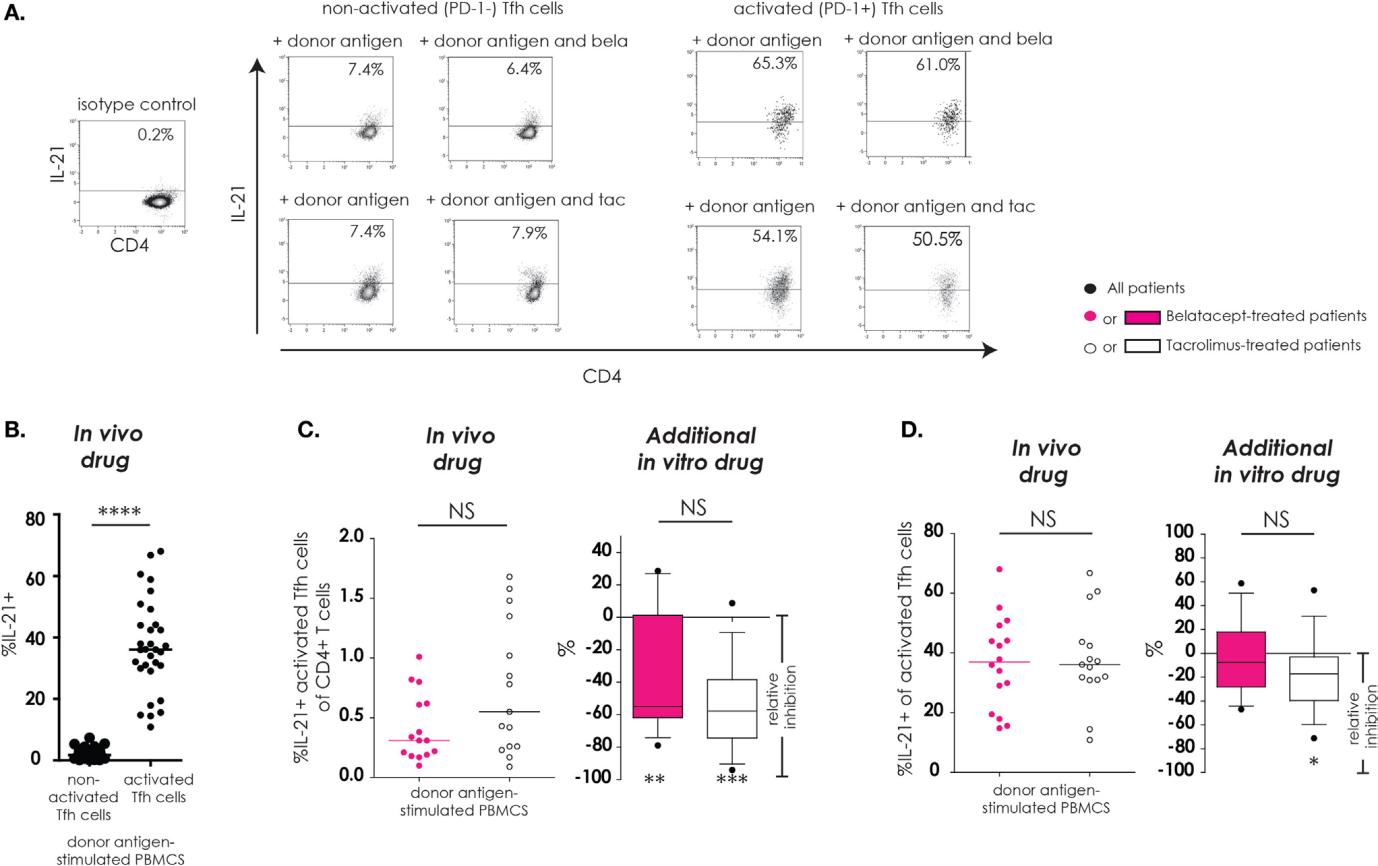
IL-21 production by remaining activated follicular T-helper (Tfh) cells was not inhibited by belatacept *in vitro*. A typical example is depicted for the intracellular IL-21 production after donor antigen stimulation in non-activated and activated Tfh-cells (CXCR5^+^PD-1^−^ and CXCR5^+^PD-1^+^ CD4^+^ T-cells, respectively) in the presence and absence of belatacept **(A)**. The proportions of IL-21^+^ cells within non-activated and activated Tfh-cells were compared after 7 days of donor antigen stimulation of peripheral blood mononuclear cells (PBMCs) obtained from both belatacept- and tacrolimus-treated patients **(B)**. The proportions of IL21^+^-activated Tfh-cells within CD4^+^ T-cells **(C)** and the proportions of IL-21^+^ cells within activated Tfh-cells **(D)** were compared between 7-day donor antigen-stimulated PBMCs obtained from the belatacept and tacrolimus group (“*in vivo*” column), as well as the relative inhibitions by *in vitro addition* of belatacept or tacrolimus (“additional *in vitro*” column). In the graphs in the “*In vivo drug*” columns, every dot represents PBMCs of a single patient. In the graphs in the “*additional in vitro drug*” columns, the relative inhibitions by *additional in vitro* belatacept and tacrolimus are depicted for aforementioned cell populations in the same belatacept- and tacrolimus-treated patients. The proportions of these cell populations after donor antigen stimulation in the absence of *in vitro* drugs are set to 0. The median relative inhibitions by belatacept and tacrolimus were tested against a theoretical median of 0. Asterisks below the boxes depict the *p*-values of these tests. The relative inhibitions were compared between belatacept and tacrolimus *in vitro*. Lines in boxes represent medians, borders of boxes represent 25th and 75th percentiles, error bars present 10th and 90th percentiles. Every box represents cultures of PBMCs obtained from *n* = 20 belatacept-treated or *n* = 20 tacrolimus-treated patients. *p < 0.05, **p < 0.01, ***p < 0.001, ****p < 0.0001, NS, not significant.

As described previously, “activated Tfh-cells” were defined as Tfh-cells that upregulated PD-1 after donor antigen stimulation and “non-activated Tfh-cells” were defined as Tfh-cells that failed to upregulate PD-1 after donor antigen stimulation. IL-21, a key cytokine in Tfh–B-cell interaction, and subsequent B-cell differentiation into immunoglobulin-producing plasma cells, was assessed in Tfh-cells (Figure 3). The donor antigen-stimulated IL-21 production was highest in activated Tfh-cells (Figure 3B). The proportions of IL21^+^-activated Tfh-cells within CD4^+^ T-cells were similar between donor antigen-stimulated PBMCs from the belatacept and tacrolimus groups (Figure 3C, “*in vivo drug*” columns). The total proportion of IL21^+^-activated Tfh-cells was partially decreased by belatacept and tacrolimus *in vitro*. The relative inhibition was −55.0% (−79.0 to +28.6%), *p* < 0.01, in the presence of belatacept and −57.7% (−94.1 to +8.7%), *p* < 0.001, in the presence of tacrolimus (Figure 3C, “*in vivo drug*” columns). No differences between the inhibition by belatacept and tacrolimus were observed (Figure 3C, “*additional in vitro drug*” column). When we focused on the remaining activated Tfh-cells in the presence of *in vitro* drugs, a substantial proportion could still produce IL-21. Even though the relative inhibitions of intracellular IL-21 production were not significantly different between belatacept and tacrolimus *in vitro*, only the latter (minimally) inhibited IL-21 production by activated Tfh-cells: relative inhibition −17.3% [−71.2 to + 52.9%, *p* < 0.05 (Figure 3D, “*additional in vitro drug*” column)].

### Summary of the Effects of Belatacept and Tacrolimus on Tfh-Cells

Belatacept and tacrolimus minimally inhibited Tfh-cell generation and partially prevented Tfh-cell activation. The proportion of IL-21^+^-activated Tfh-cells was not completely diminished by *in vitro* addition of belatacept or tacrolimus. Thus, the remaining activated Tfh-cells have the potential capacity to provide B-cell help. Next, we tested the immunosuppressive effects of both agents on B-cell activation and functional Tfh-B-cell crosstalk.

### The Effects of Belatacept and Tacrolimus on B-Cells

#### B-Cell Activation (CD86 Upregulation)

**Figure 4:**
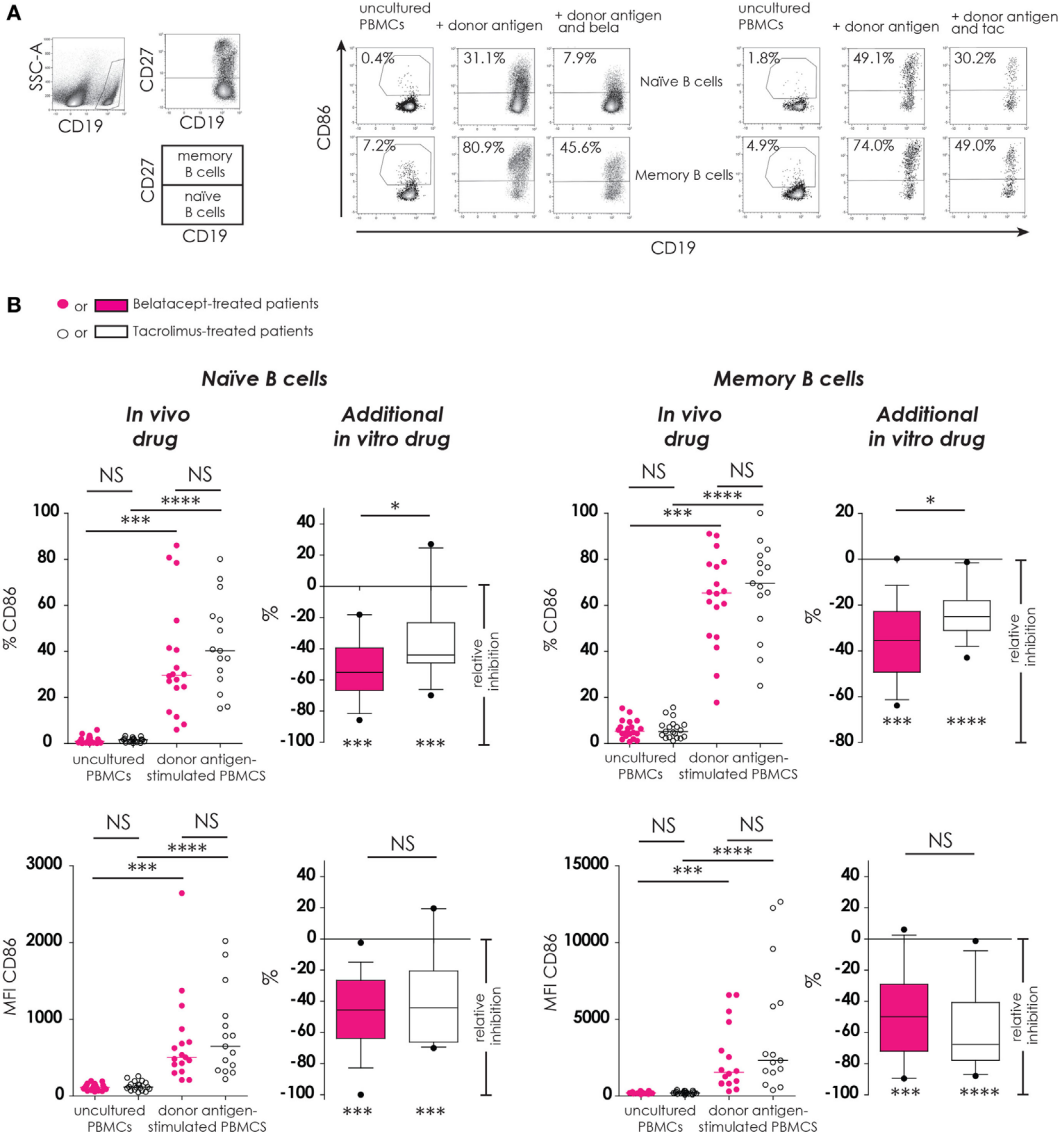
Donor antigen-stimulated CD86 upregulation on B-cells is only partially blocked by belatacept *in vitro*. Two typical examples are depicted for the free CD86 expression after 7 days of donor antigen stimulation on naïve (CD27^−^) and memory (CD27^+^) CD19^+^ B-cells, in the presence and absence of belatacept or tacrolimus **(A)**. The proportions are depicted of CD86^+^cells within naïve and memory B-cells as well as the median fluorescence intensities (MFIs) of CD86 within naïve and memory B-cells **(B)**. In the graphs in the “*In vivo drug*” columns, proportions and MFIs of aforementioned cell populations were compared (i) between belatacept- and tacrolimus-treated patients in uncultured, unstimulated PBMCs; (ii) between belatacept- and tacrolimus-treated patients in 7-day donor antigen stimulated PBMCs; (iii) between uncultured, unstimulated PBMCs and 7-day donor antigen stimulated PBMCs in belatacept-treated patients; and (iv) between uncultured, unstimulated PBMCs and 7-day donor antigen stimulated PBMCs in tacrolimus-treated patients. Every dot represents a single culture of PBMCs. In the graphs in the “*additional in vitro drug*” columns, the relative inhibitions by *additional in vitro* belatacept and tacrolimus are depicted for aforementioned cell populations in the same belatacept- and tacrolimus-treated patients. The proportions or MFIs of these cell populations after donor antigen stimulation in the absence of *in vitro* drugs are set to 0. The median relative inhibitions by belatacept and tacrolimus were tested against a theoretical median of 0. Asterisks below the boxes depict the *p*-values of these tests. The relative inhibitions were compared between belatacept and tacrolimus *in vitro*. Lines in boxes represent medians, borders of boxes represent 25th and 75th percentiles, error bars present 10th and 90th percentiles. Every box represents cultures of PBMCs obtained from *n* = 20 belatacept-treated or *n* = 20 tacrolimus-treated patients. *p < 0.05, ***p < 0.001, ****p < 0.0001, NS, not significant.

Part of the activation of B-cells and their ability to proliferate, differentiate, and function as antigen-presenting cells is reflected not only by their (free) CD86-expression but also by the expression of CD40 and ICOS-L. Here, the efficacy of belatacept was determined by means of B-cell activation, i.e., the free expression of CD86, which was measured on naïve CD19 + CD27^−^ and memory CD19^+^CD27^+^ B-cells (in proportions and MFIs), using tacrolimus as control (Figure 4). The expression of CD40 and ICOS-L on B-cells in the presence of belatacept is described in section “The Effect of Belatacept and Tacrolimus on Redundant Co-Stimulatory Pathways.”

CD86 expression was almost absent on naïve B-cells and low on memory B-cells in unstimulated uncultured PBMCs (Figure 4B, “*in vivo*” columns). No differences were observed between belatacept- or tacrolimus-treated patients. After donor antigen stimulation, both the proportions of CD86^+^ B-cells, as well as the expression of CD86 (MFIs) significantly increased on both naïve and memory B-cells (Figure 4B, “*in vivo*” columns). These were not different between the belatacept and tacrolimus group.

Despite the selective binding of belatacept to CD86 (22), the upregulation of CD86 was not completely blocked by the *in vitro* addition of belatacept. The relative inhibition of CD86 upregulation (proportion) by belatacept was −55.2% (−85.7 to −18.8%), *p* < 0.001, on naïve B-cells and −35.5% (−63.9 to +0.2%), *p* < 0.001, on memory B-cells (Figure 4B, “*additional in vitro drug*”). The relative inhibition of CD86 upregulation on naïve and memory B-cells was significantly more by belatacept than by tacrolimus *in vitro, p* < 0.05. MFIs of CD86 on naïve and memory B-cells were significantly decreased by both belatacept and tacrolimus *in vitro* (Figure 4B, “*additional in vitro drug*”). The relative inhibitions of CD86 MFIs were comparable between belatacept and tacrolimus.

To determine if the residual B-cell activation in the presence of immunosuppressive drugs was dose-dependent, the relative inhibitions by belatacept and tacrolimus were measured in the presence of supratherapeutic concentrations. Even in the presence of supratherapeutic concentrations of belatacept, membrane CD86 expression on allo-antigen-stimulated B-cells was still detectable (Figure S1 in Supplementary Material): the relative inhibition by 1,000 µg/mL belatacept (100× higher than the therapeutic concentration) was −72.4% (−86.5 to −19.7%), *p* < 0.05, in naïve B-cells and −43.2% (−53.9 to −7.4%), *p* < 0.05, in memory B-cells.

Since belatacept binds CD80 with much higher affinity than CD86 (22, 41), the residual surface expression of CD80 was low on activated naïve and memory B-cells in the presence of the different doses of belatacept (Figure S1 in Supplementary Material): The relative inhibition by 1,000 µg/mL belatacept was −90.2% (−97.5 to −75.4%), *p* < 0.05, in naïve B-cells and −85.0% (−95.1 to −57.3%), *p* < 0.05, in memory B-cells. CD80 expression on B-cells was not significantly decreased in the presence of tacrolimus.

#### B-Cell Differentiation (Plasmablast Formation)

**Figure 5:**
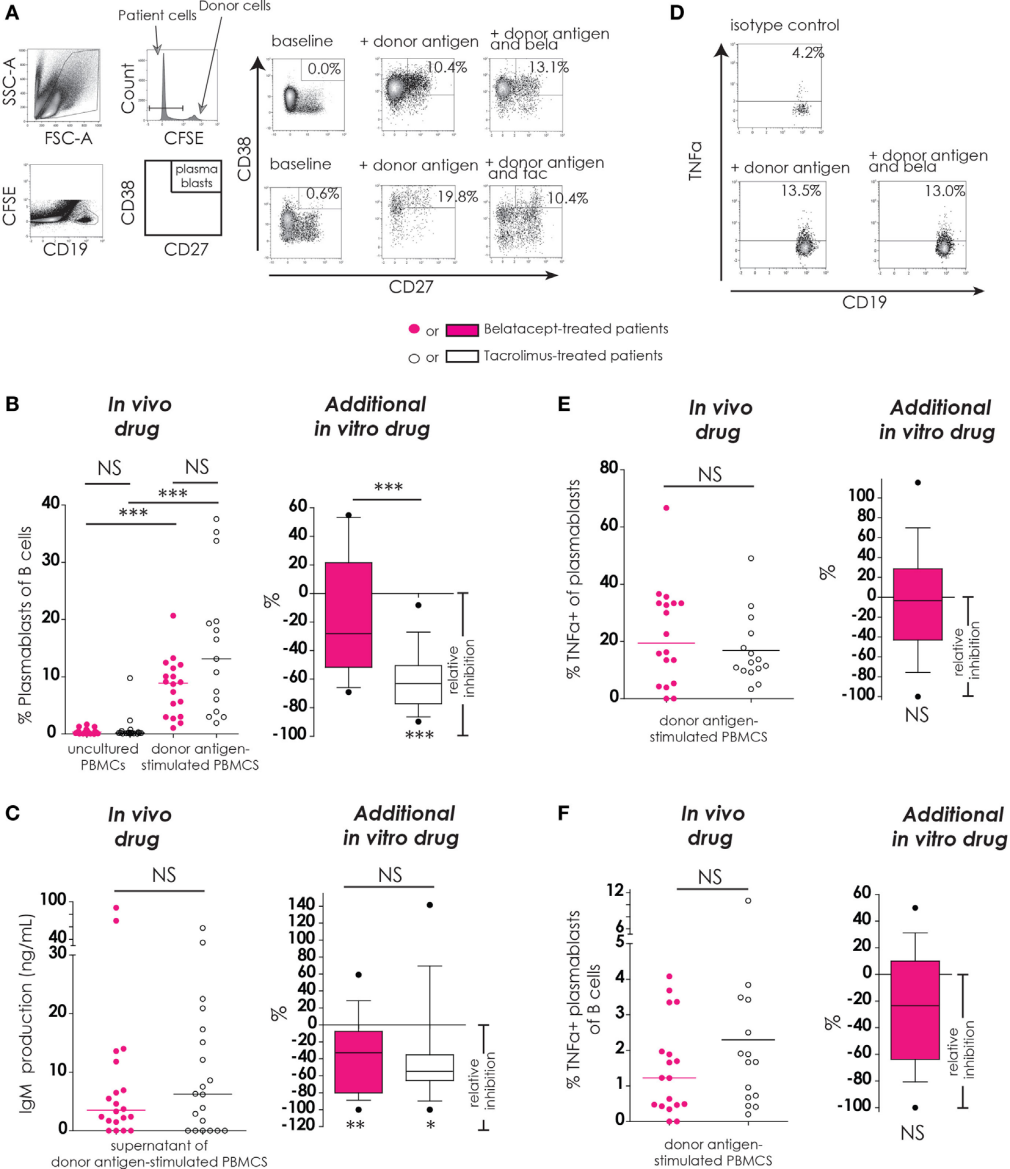
Belatacept *in vitro* did not inhibit donor antigen-driven plasmablast formation or TNFα production in a peripheral blood mononuclear cell (PBMC)-based assay, but suppressed IgM production. The gating strategy is depicted for plasmablasts (CD19^+^CD27^+^CD38^++^) after 7 days of donor antigen stimulation, in the presence or absence of belatacept and tacrolimus **(A)**. Donor PBMCs were discriminated by carboxyfluorescein succinimidyl ester labeling them prior to the mixed lymphocyte reaction and gating them out after. The proportions of plasmablasts are shown for 7-day donor antigen-stimulated PBMCs obtained from the belatacept or tacrolimus group (“*in vivo*” column), as well as the relative inhibitions by *in vitro addition* of belatacept or tacrolimus (“additional *in vitro*” column) **(B)**. The IgM concentrations in the supernatants are shown for the same cultures as previously mentioned (“*in vivo*” column), as well as the relative inhibitions by *in vitro addition* of belatacept or tacrolimus (“additional *in vitro*” column) **(C)**. A typical example is depicted for intracellular TNFα-production by plasmablasts after 7 days of donor antigen stimulation, in the presence or absence of belatacept **(D)**. The proportions of TNFα^+^ cells within plasmablasts **(E)** and the proportions of TNFα^+^ plasmablasts within B-cells **(F)** are shown for 7-day donor antigen-stimulated PBMCs obtained from the belatacept or tacrolimus group (“*in vivo*” column), as well as the relative inhibitions by *in vitro* addition of belatacept (“additional *in vitro*” column). The proportions of TNFα^+^ plasmablasts could not be reliably determined in the presence of tacrolimus *in vitro*, because of the strong inhibition of plasmablast formation by tacrolimus. In the graphs in the “*In vivo drug*” columns, every dot represents PBMCs of a single patient. In the graphs in the *“additional in vitro drug*” columns, the relative inhibitions by *additional in vitro* belatacept and tacrolimus are depicted for aforementioned cell populations in the same belatacept- and tacrolimus-treated patients. The proportions of these cell populations after donor antigen stimulation in the absence of *in vitro* drugs are set to 0. The median relative inhibitions by belatacept and tacrolimus were tested against a theoretical median of 0. Asterisks below boxes depict the *p*-values of these tests. The relative inhibitions were compared between *in vitro* belatacept and tacrolimus. Lines in boxes represent medians, borders of boxes represent 25th and 75th percentiles, and error bars present 10th and 90th percentiles. Every box represents cultures of PBMCs obtained from *n* = 20 belatacept-treated or *n* = 20 tacrolimus-treated patients. *p < 0.05, **p < 0.01, ***p < 0.001, NS, not significant. N.B.: The median fluorescence intensity slightly decreases in (antigen-) stimulated cells compared to unstimulated cells, partly because of the intracellular staining protocol that was used to determine intracellular cytokine expression. Therefore, the gates in the unstimulated and stimulated cells are not exactly the same.

To study the effect of belatacept and tacrolimus on the antigen-dependent Tfh–B-cell interaction, differentiation of B-cells into plasmablasts was measured in donor antigen-activated PBMCs obtained after transplantation (Figure 5).

The proportions of plasmablasts were equally low in PBMCs from belatacept- and tacrolimus-treated patients (Figure 4B, “*in vivo drug*” column). Plasmablast formation was significant after donor antigen stimulation in PBMCs from the belatacept group [8.8% (1.0–20.7%), *p* < 0.001] and from the tacrolimus group [13.1% (1.9–37.6%), *p* < 0.001], belatacept vs. tacrolimus group, *p* = 0.10. Only tacrolimus significantly inhibited plasmablast formation with a relative inhibition of −50.5% (−89.7 to −8.2%), *p* < 0.0001 (Figure 5B, “*additional in vitro drug*” column). Belatacept failed to inhibit this alloreactive process in PBMCs, and its relative inhibition [−28.1% (−69.1 to +54.8%)] was significantly less than the inhibition by tacrolimus, *p* < 0.001.

#### Plasmablast Function (IgM Production and Intracellular TNFα Production)

IgM production by donor antigen-stimulated PBMCs was not significantly different in PBMCs obtained from the belatacept-treated patients compared to the tacrolimus-treated patients (Figure 5C, “*in vivo drug*” column). The relative inhibitions of IgM production by belatacept and tacrolimus *in vitro* were −32.9% (−100.0 to +59.1%), *p* < 0.01, and −54.9% (−100.0 to +141.4%), *p* < 0.05, respectively (Figures 5C, “*additional in vitro drug*” column). Even though tacrolimus more efficiently inhibited plasmablast formation than belatacept, the inhibition of IgM production did not significantly differ between these two drugs.

Since belatacept is a fusion protein consisting of the Fc-fragment of IgG1 (22), total human IgG could not be determined by ELISA. No IgG DSA were detected by Luminex in supernatants of the MLRs. IgG3 could be detected in eight cultures with donor-antigen stimulated PBMCs [median 6.6 (4.8–4.3) ng/mL; 5× from tacrolimus-treated and 3× from belatacept-treated patients] and was −12.3% (−79.3 to + 33.5%) inhibited in these samples by tacrolimus or belatacept, *p* = 0.01 (Figure S2 in Supplementary Material). Because of the limited amount of IgG3^+^ supernatants no subgroup analysis per treatment arm was performed.

Of the B-cells that differentiated into plasmablasts 19.4% (0.0–66.7%) expressed intracellular TNFα in the PBMCs obtained from belatacept-treated patients and 12.7% (3.4–49.1%) in those from the tacrolimus-treated patients, *p* = 0.34 (Figure 5E, “*in vivo*” column). The proportions of TNFα^+^ plasmablasts within total B-cells were also similar in PBMCs from belatacept- and tacrolimus-treated patients: 1.2% (0.0–4.1%) and 1.7% (0.2–10.1%), respectively, *p* = 0.34 (Figure 5F, “*in vivo*” column). Belatacept did not affect the proportion of TNFα^+^ within plasmablasts nor the proportions of TNFα^+^ plasmablasts within total B-cells (Figures 5E,F, “*additional in vitro*” column). The proportions of TNFα^+^ plasmablasts could not be reliably determined in the presence of tacrolimus, because of the strong inhibition of plasmablast formation by tacrolimus *in vitro*.

#### B-Cell Differentiation and Plasmablast Function in an Isolated Coculture System

To eliminate the effects of other cell types and cytokines present in the PBMC-based assay, we tested the effects of belatacept and tacrolimus in an isolated system of antigen-activated CXCR5^+^ Tfh and CD19^+^CD27^+^ memory B-cells (Figure S3 in Supplementary Material). The differentiation of memory B-cells into IgM producing plasmablasts was used as read out. Plasmablast formation of 13.2% (2.1–44.9%) was decreased by the addition of belatacept to 1.7% (1.3–4.2%), and by tacrolimus to 0.5% (0.1–0.9%), both *p* < 0.05 (Figure S3D in Supplementary Material). Tacrolimus more potently inhibited the plasmablast formation than belatacept, *p* < 0.05. The same pattern was seen in the IgM production of 253.8 ng/mL (86.5–541.5 ng/mL) (Figure S3E in Supplementary Material). Belatacept decreased IgM production to 18.1 ng/mL (13.7–68.4 ng/mL), and tacrolimus to 6.2 ng/mL (2.2–8.6 ng/mL) (both *p* < 0.05; tacrolimus vs. belatacept *p* < 0.05).

#### Immune Regulatory Phenotype (IL-10^+^ Transitional B-Cell Survival)

**Figure 6:**
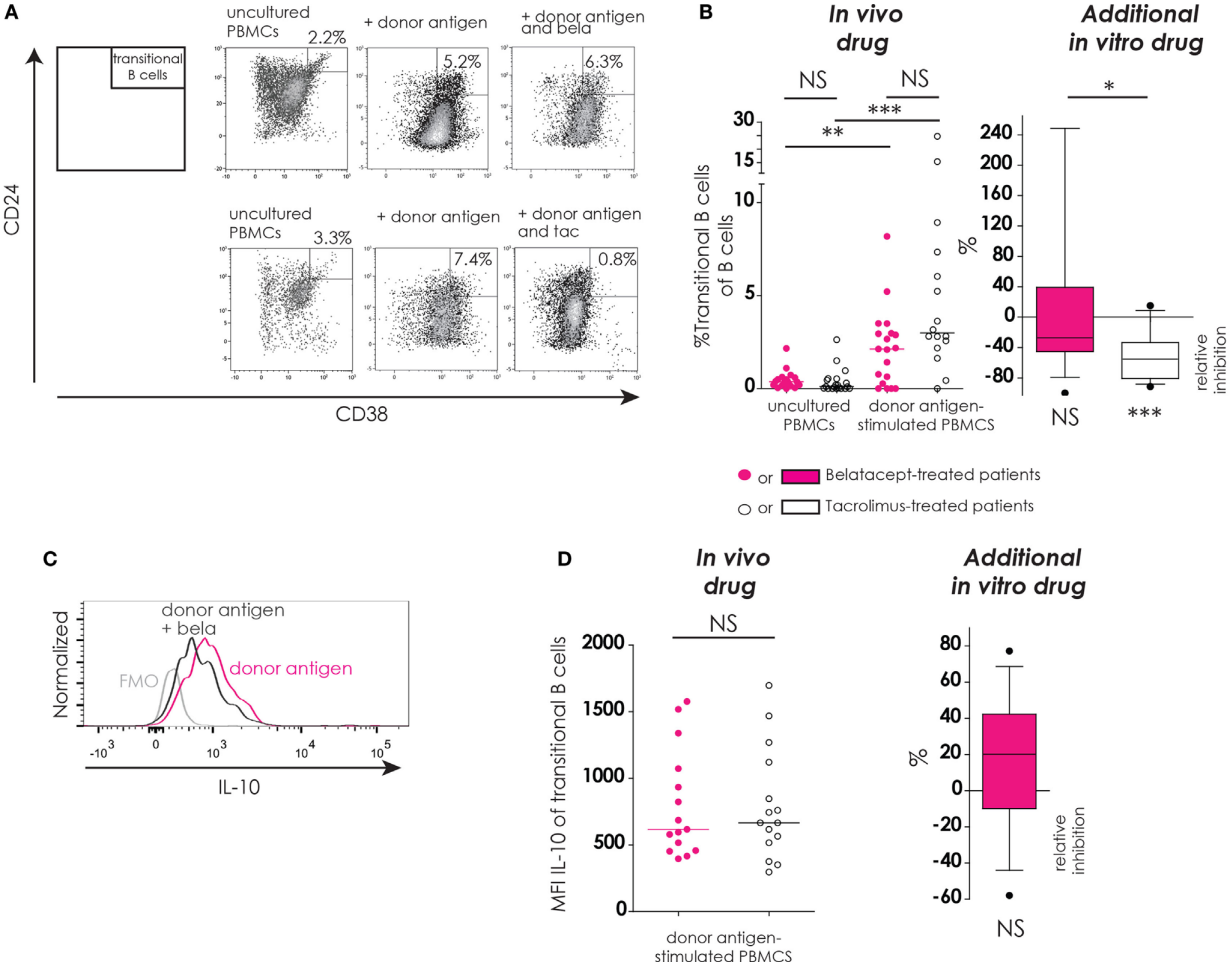
Transitional B-cells and their donor antigen-driven IL-10 production were conserved by belatacept *in vitro* but inhibited by tacrolimus. The gating strategy is depicted for transitional B-cells (CD24^+^CD38^++^) after donor antigen stimulation **(A)**. Cells were gated from CD19^+^ B-cells like depicted in Figure 4A. The proportions of transitional B-cells are shown for 7-day donor antigen-stimulated peripheral blood mononuclear cells (PBMCs) obtained from the belatacept or tacrolimus group (“*in vivo*” column), as well as the relative inhibitions by *in vitro addition* of belatacept or tacrolimus (“additional *in vitro*” column) **(B)**. A typical example is depicted for intracellular IL-10 expression [median fluorescence intensity (MFI)] by transitional B-cells after 7 days of donor antigen stimulation, in the presence or absence of belatacept, including a Fluorescence-Minus-One control (FMO) **(C)**. The MFIs of IL-10 within transitional B-cells are shown for 7-day donor antigen-stimulated PBMCs obtained from the belatacept or tacrolimus group (“*in vivo*” column), as well as the relative inhibitions by *in vitro addition* of belatacept (“*additional in vitro*” column) **(D)**. The MFI of IL-10 within transitional B-cells could not be reliably determined in the presence of tacrolimus *in vitro*, because of the decreased transitional B-cells survival in the presence of tacrolimus. In the graph in the “*In vivo drug”* column in **(B)**, proportions of transitional B-cell populations were compared (i) between belatacept- and tacrolimus-treated patients in uncultured, unstimulated PBMCs; (ii) between belatacept- and tacrolimus-treated patients in 7-day donor antigen stimulated PBMCs; (iii) between uncultured, unstimulated PBMCs and 7-day donor antigen stimulated PBMCs in belatacept-treated patients; and (iv) between uncultured, unstimulated PBMCs and 7-day donor antigen stimulated PBMCs in tacrolimus-treated patients. Every dot represents PBMCs of a single patient. In the graphs in the “*Additional in vitro drug*” column the relative inhibitions by *additional in vitro* belatacept and tacrolimus are depicted for aforementioned cell populations in the same belatacept- and tacrolimus-treated patients. The proportions of these cell populations after donor antigen stimulation in the absence of *in vitro* drugs are set to 0. The median relative inhibitions by belatacept and tacrolimus were tested against a theoretical median of 0. Asterisks below boxes depict the *p*-values of these tests. The relative inhibitions were compared between *in vitro* belatacept and tacrolimus. Lines in boxes represent medians, borders of boxes represent 25th and 75th percentiles, error bars present 10th and 90th percentiles. Every box represents cultures of PBMCs obtained from *n* = 20 belatacept-treated or *n* = 20 tacrolimus-treated patients. *p < 0.05, **p < 0.01, ***p < 0.001, ****p < 0.0001, NS, not significant. N.B.: The MFI slightly decreases in antigen-stimulated cells compared to unstimulated cells, partly because of the intracellular staining protocol that was used to determine intracellular cytokine expression. Therefore, the gates in the unstimulated and stimulated cells are not exactly the same.

The presence of B-cells with a regulatory phenotype, i.e., IL10^+^ transitional B-cells, was assessed after donor antigen stimulation in the presence or absence of belatacept or tacrolimus (Figure 6).

In unstimulated, uncultured PBMCs the proportions of transitional B-cells were below 3% in both treatment groups (Figure 5B, “*in vivo drug*” column). After donor antigen stimulation, an increase in the proportion of transitional B-cells was observed in PBMCs from belatacept- and tacrolimus-treated patients, to 2.1% (0.0–8.2%), *p* < 0.01, and 3.0% (0.0−24.7%), *p* < 0.001, respectively (Figure 5B, “*in vivo drug*” column). The survival of these transitional B-cells was not different between the belatacept and tacrolimus groups. The *in vitro* addition of tacrolimus, however, diminished transitional B-cell survival [relative inhibition: −55.4% (−91.4 to −15.1%), *p* < 0.001], while belatacept did not affect the survival of these potentially regulatory B-cells [relative inhibition: −27.2% (−100.0 to +247.4%), *p* = 0.54], tacrolimus vs. belatacept, *p* < 0.05.

Both the transitional B-cells in the PBMCs obtained from belatacept-treated patients as those from tacrolimus-treated patients expressed IL-10 after donor antigen stimulation: MFI 617 (397 to 1577) and MFI 666 (297 to 1697), respectively, *p* = 1.00 (Figure 6D, “*in vivo*” column). Belatacept *in vitro* did not decrease IL-10 production in transitional B-cells (Figure 6D, “*additional in vitro*” column). The MFI of IL-10 within transitional B-cells could not be reliably determined in the presence of tacrolimus, because of the decreased transitional B-cells survival in the presence of tacrolimus *in vitro*.

#### Summary of the Effects of Belatacept and Tacrolimus on B-Cells

Donorantigen-driven CD86 upregulation was not fully inhibited by belatacept, especially on memory B-cells, even by supratherapeutic doses of belatacept. In contrast to tacrolimus, belatacept could not inhibit donorantigen-driven plasmablast formation in a PBMC-based assay but only in an isolated Tfh-B-cell coculture. Also in the latter, belatacept was less effective than tacrolimus. The survival of the potentially immune regulatory IL-10^+^ transitional B-cells was, however, not affected by belatacept, while this was diminished by tacrolimus.

### Redundancy of the Immune System

#### The Effect of Belatacept and Tacrolimus on Redundant Co-Stimulatory Pathways

To explain why belatacept did not inhibit plasmablast formation in our PBMC studies, and because patients’ cellular interactions are influenced by redundant and pleiotropic mechanisms of immune cells, surface receptors of other costimulatory pathways were measured (Figure S4 in Supplementary Material).

In PBMCs from three belatacept-treated and three tacrolimus-treated patients, the ICOS-ICOSL, PD-1-PD-L1, and the CD40L-CD40 pathways were studied after donor antigen stimulation Expressions of all surface molecules, except for CD28 and ICOSL, were increased on Tfh and B-cells after donor antigen stimulation (Figures S4A,C in Supplementary Material). The upregulation of the costimulatory molecules on Tfh-cells was not fully suppressed by belatacept and to a lesser extent than by tacrolimus (Figure S4 in Supplementary Material).

### Multivariable Regression Analyses

#### The Effect of Belatacept and Tacrolimus *in Vitro* on Tfh- and B-Cells

The effect of belatacept *in vitro* was compared to the effect of tacrolimus *in vitro* in multivariable regression analyses for proportions of CD86^+^ naïve and memory B-cells, plasmablast formation, and transitional B-cell survival (adjusted for the variables as stated in Table 1). These cell subsets were chosen, because they significantly differed when belatacept was added compared to tacrolimus *in vitro* (Figures 4–6). Multivariable analyses confirmed that belatacept and tacrolimus differed in inhibition of plasmablast formation: plasmablast formation was 4.5% (SE 1.3) higher in the presence of belatacept than in the presence of tacrolimus *in vitro, p* = 0.001 (Table 1). In the multivariable analysis, transitional B-cell survival (defined as the proportion of transitional B-cells of total B-cells) was not significantly higher in the presence of belatacept compared to *in vitro* addition of tacrolimus (*p* = 0.91). Finally, the free CD86 expression on CD27^+^ memory B-cells was 9.3% (SE 2.8) lower when the donor antigen-stimulated PBMCs were spiked with belatacept *in vitro* compared to tacrolimus *in vitro, p* = 0.001, but no significant difference was found for free CD86-expression on the surface of naïve B-cells (*p* = 0.12).

**Table 1:** The effect of belatacept *in vitro* on free CD86 expression on B-cells, plasmablast formation, and transitional B-cell survival.

Dependent variable	Independent variables	Beta	SE	*p*
CD86 expressing naïve B-cells (% CD86^+^ of CD19^+^CD27^−^ B-cells)	Belatacept added *in vitro* (vs. tacrolimus added *in vitro*)	−4.53	2.85	0.12
	PBMCs obtained after transplantation (vs. before transplantation)	1.76	2.50	0.48
	PBMCs obtained from rejector (vs. non-rejector)	−0.09	3.02	0.98
	Proportion of cell subset without *in vitro* drugs added	0.55	0.06	0.00

CD86 expressing memory B-cells (% CD86^+^ of CD19^+^CD27^+^ B-cells)	Belatacept added *in vitro* (vs. tacrolimus added *in vitro*)	−9.34	2.84	0.02
	PBMCs obtained after transplantation (vs. before transplantation)	−1.15	2.49	0.65
	PBMCs obtained from rejector (vs. non-rejector)	2.10	3.01	0.49
	Proportion of cell subset without *in vitro* drugs added	0.61	0.07	0.00

Plasmablasts (% CD27^+^CD38^++^ of CD19^+^ B-cells)	Belatacept added *in vitro* (vs. tacrolimus added *in vitro*)	4.45	1.28	0.00
	PBMCs obtained after transplantation (vs. before transplantation)	−1.57	1.08	0.15
	PBMCs obtained from rejector (vs. non-rejector)	2.34	1.32	0.08
	Proportion of cell subset without *in vitro* drugs added	0.66	0.07	0.00

Transitional B-cells (% of CD19^+^ B-cells)	Belatacept added *in vitro* (vs. tacrolimus added *in vitro*)	−0.09	−0.01	0.91
	PBMCs obtained after transplantation (vs. before transplantation)	−0.38	0.71	0.59
	PBMCs obtained from rejector (vs. non-rejector)	0.40	0.86	0.65
	Proportion of cell subset without *in vitro* drugs added	0.24	0.07	0.00

*A multivariable regression analysis was performed per cell subset with as dependent variable the value after donor antigen stimulation plus *in vitro* drugs (belatacept or tacrolimus) and as independent variables addition of *in vitro* belatacept (vs*. in vitro* tacrolimus), the time point PBMCs were obtained (after vs. before transplantation), PBMCs obtained during rejection (rejector vs. non-rejector) and the value after donor antigen stimulation without *in vitro* drugs added. Statistically significant *p*-values (<0.05) are underscored*.

*Proportions of the different donor antigen-stimulated subsets without *in vitro* drugs added were most predictive for the proportions in the presence of *in vitro* drugs. In 11 belatacept-treated patients and 2 tacrolimus-treated patients the PBMCs were obtained during acute rejection, before additional anti-rejection therapy was given. Obtaining PBMCs from patients who rejected or time point the PBMCs were obtained (before or after transplantation) were not predictive for the proportions of these cells*.

*PBMCs, peripheral blood mononuclear cells; Rejector vs. non-rejector, patients who rejected vs. patients who did not reject within 12 months after transplantation (biopsy-proven); SE, standard error of beta*.

Peripheral blood mononuclear cells were obtained 3 months after transplantation in non-rejectors, and during rejection before additional anti-rejection therapy was given in rejectors. Eleven of twenty belatacept-treated patients and two of twenty tacrolimus-treated patients developed a BPAR. Obtaining PBMCs during acute rejection did not alter the *in vitro* reaction to donor antigen or drug (Table 1).

The effects of belatacept and tacrolimus *in vitro* on the different Tfh- and B-cell subsets are summarized in Table 2.

**Table 2:** Effects of belatacept and tacrolimus *in vitro* on donor antigen-activated follicular T-helper (Tfh) and B-cells.

Immunological reaction	Defined by	Effect by belatacept *in vitro*	Effect by tacrolimus *in vitro*	Comparison belatacept vs. tacrolimus^a^
Tfh-cell generation	CXCR5 expression ↑ on CD4^+^ T-cells	Inhibition (minimal)	Inhibition (minimal)	bela = tac
Tfh-cell activation	PD-1 expression ↑ on CD4^+^CXCR5^+^ T-cells	Inhibition (partial)	Inhibition (partial)	bela = tac
Activated Tfh-cell generation	CXCR5^+^PD-1^+^ double expression ↑ on CD4^+^ T-cells	Inhibition (partial)	Inhibition (partial)	bela = tac
IL-21^+^-activated Tfh-cell generation	The proportion of IL-21^+^ CXCR5^+^PD-1^+^ ↑ within CD4^+^ T-cells	Inhibition (partial)	Inhibition (partial)	bela = tac
IL-21 production by activated Tfh-cells	Intracellular IL-21 expression of CXCR5^+^PD-1^+^ CD4^+^ T-cells	None	Inhibition (minimal)	bela = tac
B-cell activation	CD86 expression ↑ on B-cells	Inhibition (partial)	Inhibition (partial)	bela is more efficient than tac^b^
Transitional B-cell survival	The proportion of CD24^+^CD38^++^ B-cells	None	Inhibition (partial)	bela is more benificial than tac^c^
Plasmablast formation	Proportion of B-cells differentiated into CD27^+^CD38^++^ B-cells	*In PBMCs*: none	*In PBMCs*: inhibition (partial)	bela is less efficient than tac^d^
		*In isolated system with Tfh- and B-cells*: inhibition (almost completely)	*In isolated system with Tfh- and B-cells*: inhibition (completely)	bela is slightly less efficient than tac
IgM production	Total IgM in supernatant of cocultures	*In PBMCs*: inhibition (partial)	*In PBMCs*: inhibition (partial)	bela = tac
		*In isolated system with Tfh- and B-cells*: inhibition (almost completely)	*In isolated system with Tfh- and B-cells*: inhibition (completely)	bela is slightly less efficient than tac

*bela, belatacept; IgM, immunoglobulin M; PBMCs, peripheral blood mononuclear cells; tac, tacrolimus*.

*^a^Comparison of the relative inhibition by belatacept in vitro and tacrolimus in vitro*.

*^b^These observations were confirmed in a multivariable regression analysis for memory B-cells, but not for naïve B-cells (Table 1)*.

*^c^These observations were not confirmed in a multivariable regression analysis (Table 1)*.

*^d^These observations were confirmed in a multivariable regression analysis (Table 1)*.

## Discussion

In this study, the effects of belatacept on Tfh–B-cell interaction were compared to those of tacrolimus for the first time in kidney transplant patients. No differences were observed in unstimulated uncultured PBMCs or donor antigen-stimulated PBMCs obtained from belatacept- or tacrolimus-treated patients, which may be explained by the predominant effects by MMF and prednisone in both regimens. Therefore, the isolated effects of *in vitro* belatacept and tacrolimus were compared. *In vitro* addition of both drugs only minimally inhibited Tfh-cell generation and partially decreased activation of Tfh-cells (defined by PD-1 upregulation). Activated Tfh-cells produced the highest levels of IL-21, and the total proportion of IL-21^+^ activated Tfh-cells in the presence of *in vitro* immunosuppression was also partially reduced. Still, IL-21 production and B-cell help by remaining Tfh-cells were sufficient in the presence of *in vitro* belatacept, because the donor antigen-driven formation of plasmablasts in our MLR-based PBMC assay was not inhibited by the costimulation blocker, in contrast to *in vivo* observations in animal studies (14, 23). These newly formed TNFα^+^ plasmablasts, that have been associated with aggressive reactivity in autoimmunity (42), were suppressed in the presence of tacrolimus.

A first explanation for these findings is the differences between our study and previous work (14, 23, 24). Belatacept has always been compared to CsA and not with the more potent tacrolimus and used in combination with other types of immunosuppressive agents, such as T-cell depleting therapy or mTOR inhibition in the animal studies (14, 23) or MMF and prednisone in the BENEFIT trial (24). The study presented here reports on the isolated effects of belatacept and tacrolimus on the functional interaction of patient-derived Tfh- and B-cells. These differences might have led to an overestimation of the inhibition of Tfh–B-cell interaction by belatacept, not taking into account the effects of other immunosuppressive agents.

A second reason could be the significant residual expression of CD86 on donor antigen-activated B-cells, even in the presence of supratherapeutic concentrations of belatacept. This might be explained by (1) a lower affinity of belatacept for donor antigen-activated CD86 molecules on B-cells, (2) a higher turnover of CD86 by B-cells, or (3) degradation of belatacept during the 7-day cultures. The latter is unlikely, since CD80 was efficiently blocked by belatacept. Until the study presented here, the efficacy of belatacept on occupying CD86 had only been studied on monocyte-derived dendritic cells (DCs) and not on B-cells (41). As a result of the incomplete blockade of CD86 on B-cells, activation and consequently differentiation of B-cells were not prevented by costimulation blockade. The production of IgM was, however, inhibited by belatacept (median ~50%), possibly because CD80 blockade or partial CD86 blockade also leads to impaired immunoglobulin responses (43, 44). Nonetheless, belatacept was not more efficient than tacrolimus in preventing IgM production and even slightly less efficient in an isolated system. The lower percentage of DSA-positive patients in the belatacept than in the CsA group in the BENEFIT trial (24) could be (1) a reflection of better compliance in the first group (45, 46), (2) the lower potency of CsA compared to tacrolimus (25), and (3) higher concentrations of mycophenolate acid in the first group (47, 48).

A third answer can be found in redundant costimulatory pathways taking over during costimulation blockade of the CD28-CD80/86 pathway. Because belatacept affects only this pathway (22), other costimulatory pathways, such as CD40-CD40L and ICOS-ICOSL, may “bypass” blockade of CD28-CD80/86. In our small cohort study of *n* = 6 independent experiments, the upregulation of CD40L and ICOS on Tfh-cells were less reduced by belatacept than by tacrolimus, making these cells more capable of helping B-cells. Since tacrolimus has a direct effect on T- and B-cells by inhibiting calcineurin downstream the surface receptors (27, 29, 49), its effect is not dependent on costimulation blockade. Further studies that test the combination of belatacept with CD40- or ICOS-blockade could confirm this hypothesis but were beyond the scope of the study presented here.

A final possibility is that belatacept less effectively inhibits DCs and their interaction with Tfh- and B-cells than the interaction between Tfh- and B-cells, especially in an *in vitro* setting in the absence of a germinal center (50–52). Unlike in donor antigen-stimulated PBMCs, in an isolated system of pure CXCR5^+^ Tfh and memory B-cells, belatacept successfully inhibited plasmablast formation. A big difference between PBMCs and isolated Tfh and memory B-cells is the absence of patient DCs and their antigen-presenting function in the isolated system, i.e., the absence of the indirect and semidirect pathways of antigen presentation (53). The effect of belatacept on human DCs is not yet studied and a lack hereof could explain the less efficient inhibition by belatacept on Tfh–B-cell interaction. Nevertheless, donor DCs, facilitating the direct pathway for antigen presentation, were still present in the isolated system. This suggests belatacept effectively inhibits the direct, but not the indirect or semidirect pathways of antigen presentation. Absence of other cells, like natural killer cells, could also be an explanation for the successful inhibition by belatacept in the isolated system. We believe a PBMC-based assay is more similar to the milieu in patients than an isolated cell assay, because in the first system, multiple cell types and redundant pathways are of importance.

It should be noted almost no IgG3 by ELISA and no anti-HLA IgGby Luminex were detected in the culture supernatants, possibly because only materials of immunologically low-risk patients were used. Since belatacept is an IgG1, it cannot be ruled out that IgG1 antibodies were present in the cocultures’ supernatants. Another limitation of this study is that anti-CD86 monoclonal antibodies that are non-competitive to belatacept and bind to another epitope than belatacept are not commercially available (41). The total CD86 expression, irrespective of saturated CD86 by belatacept in the cocultures, could therefore not be determined.

A possible beneficial consequence of the incomplete inhibition of B-cells by costimulation blockade is that belatacept favored the (potentially regulatory) IL-10^+^ transitional B-cell survival, while this was diminished by tacrolimus (30–32). However, these findings were not confirmed by multivariable regression analyses. Therefore, the clinical relevance of these observations needs to be validated in a larger population. Even more, since (1) most rejections occur within the first months after transplantation, when glucocorticoids are still used and negatively influence transitional B-cell survival (30) and (2) the regulatory capacities of antigen-specific transitional B-cells have not yet been confirmed in functional studies in humans. Another favorable outcome of incomplete Tfh-B-cell inhibition by belatacept could be a lower infection risk and more potent vaccine responses in belatacept-treated patients than in tacrolimus-treated patients. So far no evidence for this emerged from previous studies nor has it been confirmed in randomized-controlled trials comparing belatacept and CNI-treated patients (24, 54–56).

In this functional study, belatacept was less potent than tacrolimus in inhibiting donor antigen-driven plasmablast formation.

## Ethics Statement

Materials were collected from 40 kidney transplant patients and their donors who participated in a prospective, randomized-controlled trial (approved by the Medical Ethical Committee of the Erasmus MC, University Medical Centre Rotterdam; MEC-2012-42, EUDRACT CT # 2012-003169-16). After written informed consent, patients were included and randomized to a tacrolimus-based (control) or belatacept-based (experimental) immunosuppressive regimen. All procedures were in accordance with the ethical standards of the Declaration of Istanbul (International Summit on Transplant and Organ, 2008).

## Author Contributions

GG designed, conducted and analyzed experiments, and wrote the manuscript; DH, WW, and CC designed experiments and edited the manuscript; MD, RK, WV, and NL designed, analyzed, and/or conducted experiments; and DR and AS interpreted data and edited the manuscript.

## Conflict of Interest Statement

Despite funding from pharmaceutical companies (see “Funding”), the authors declare that this research was conducted and presented in the absence of any conflict of interest.
