# Supplementary material for: Belatacept Does Not Inhibit Follicular T Cell-Dependent B-Cell Differentiation in Kidney Transplantation
Source: Front Immunol. 2017 May 31;8:641. doi: 10.3389/fimmu.2017.00641 (PMC5450507; doi:10.3389/fimmu.2017.00641)
Supplement: Supplementary file 1 [file image_1.pdf]

Supplementary Figures

Belatacept Does Not Inhibit Follicular T Cell-Dependent B-Cell Differentiation in Kidney Transplantation

Gretchen N. de Graav[*], Dennis A. Hesselink[*], Marjolein Dieterich[*], Rens Kraaijeveld[*], W. Verschoor[*], Dave L. Roelen[†], Nicolle H.R. Litjens[*], Anita S. Chong[‡], Willem Weimar[*], Carla C. Baan[*]

[*]Department of Internal Medicine, Section Transplantation and Nephrology, Erasmus MC, University Medical Center, Rotterdam, the Netherlands; [†]Department of Immunohematology and Blood Transfusion, Leiden University Medical Center, Leiden, the Netherlands; [‡]Department of Surgery, The University of Chicago, Chicago, Illinois, The United States of America

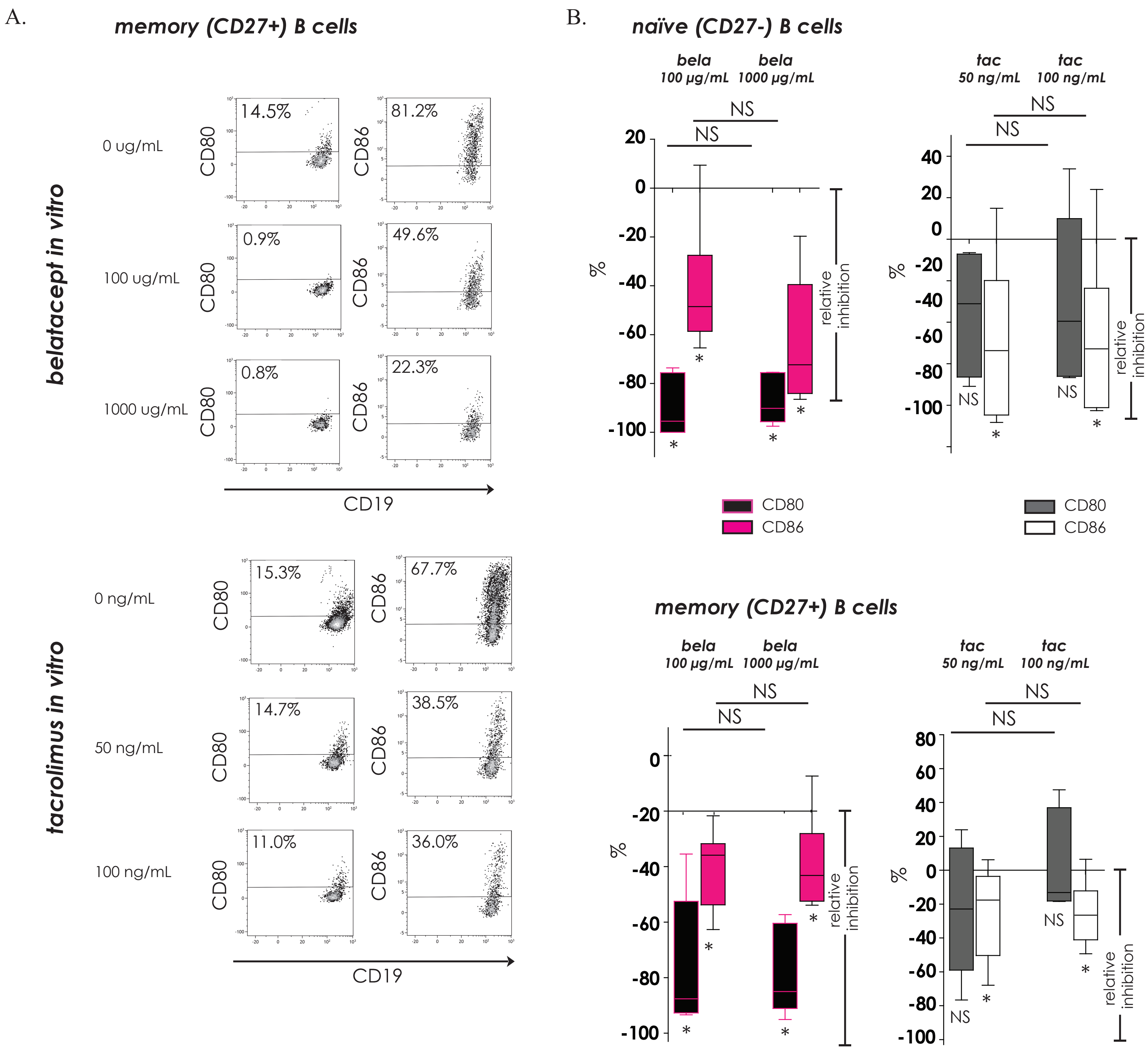

Supplementary Figure 1. CD86 is not fully blocked by belatacept on alloantigen-activated B-cells, using 100x higher than therapeutic concentrations.

A typical example is shown for free CD80 and CD86 expression on memory B-cells after allo-antigen stimulation in the presence of two supra-therapeutic concentrations of belatacept and tacrolimus (A). The expression on naïve B-cells was gated in the same way. The relative inihibitions of CD80 and CD86 on naïve (CD27-) and memory (CD27+) CD19+ B-cells after allo-antigen stimulation is depicted for 6 independent mixed lymphocyte reactions using peripheral blood mononuclear cells of healthy controls (B). Allogeneic PBMCs were CD19-depleted before adding them to the cultures. The expression of CD80 and CD86 without drugs is set to zero.

N.B.:

Lines in boxes represent medians, borders of boxes represent 25th and 75th percentiles, error bars present 10th and 90th percentiles. Every box represents cultures of PBMCs obtained from n = 6 healthy controls. Using the Wilcoxon Signed Rank test, the median relative inhibitions by belatacept and tacrolimus were tested against a theoretical median of 0. Asterisks below boxes depict the p-values of these tests. The relative inhibitions between the different concentrations were also compared using the Wilcoxon Signed Rank test.

* = $p<0.05$ / ** = $p<0.01$ / *** = $p<0.001$ / **** = $p<0.0001$ / NS = not significant

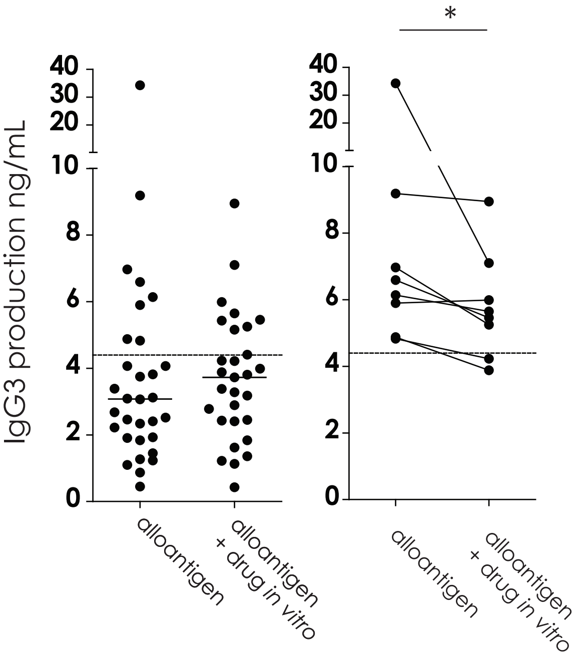

Supplementary Figure 2. IgG3 production after 7 days of donor-antigen stimulation of PBMCs obtained after kidney transplantation.

The calibration curve of the IgG3 ELISA started at 4.4 ng/mL (dotted line). IgG3 concentration was above this cut-off point in 8 cultures with donor-antigen stimulated PBMCs: 5x from tacrolimus-treated and 3x from belatacept-treated patients), and was inhibited in all these samples by tacrolimus or belatacept (right column). Because of the limited amount of IgG3+ supernatants no subgroup analysis per treatment arm was performed.

N.B.: * $p<0.05$

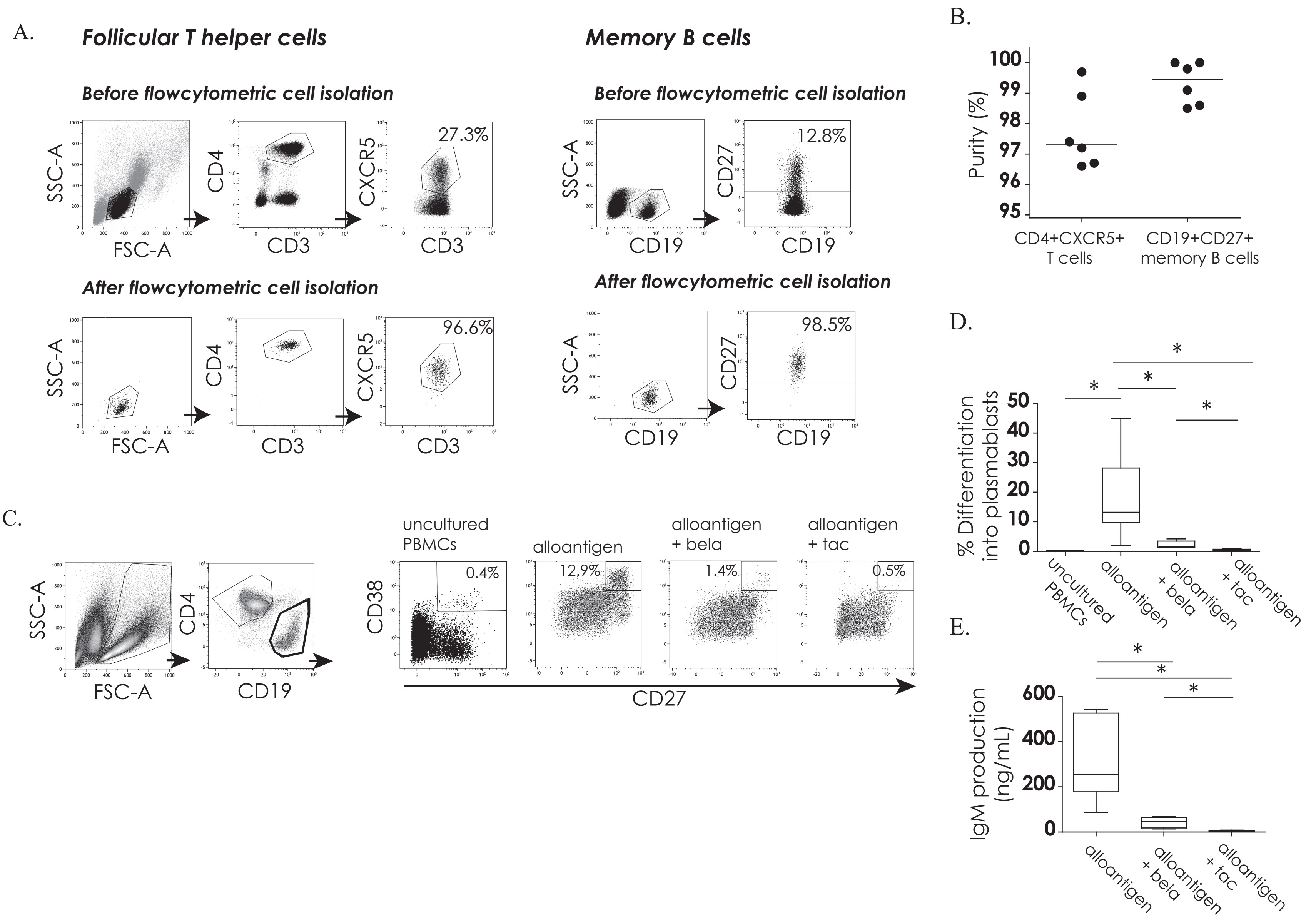

Supplementary Figure 3. Belatacept inhibited plasmablast formation and IgM production in an allo-antigen activated co-culture of isolated follicular T helper (Tfh) cells and memory B-cells
A typical example of Tfh cells and memory B-cells is depicted before and after flow cytometric cell isolation (A). The purities are depicted of the isolated CD4+CXCR5+ T-cells and CD19+CD27+ memory B-cells in 6 independent experiments, using materials of 3 healthy controls and 3 patients before transplantation (B). A typical example is shown for CD27+CD38++ plasmablasts after 7 days of alloantigen stimulation in a co-culture of isolated CD4+CXCR5+ T helper cells and CD19+CD27+ memory B-cells, in the presence or absence of belatacept 10 µg/mL or tacrolimus 10 ng/mL (C). Allogeneic PBMCs were CD3/19-depleted and irradiated (40 Gy) before adding them to the cultures. The proportions of plasmablasts are shown for uncultured PBMCs, and alloantigen-stimulated isolated CD19+CD27+ memory B-cells and CD4+CXCR5+ T helper cells, in the presence or absence of belatacept 10 µg/mL or tacrolimus 10 ng/mL (D). For the experiments with belatacept and tacrolimus the same materials were used, namely of 3 healthy controls and 3 pre-transplant patients. The IgM concentration in the supernatants are shown for above mentioned co-cultures (E).

N.B.: The black lines in the boxes represent the medians. The upper and lower borders of the boxes represent the 25th and 75th percentile. The error lines represent the 10th and 90th percentiles. bela=belatacept 10 µg/mL, tac=tacrolimus 10 ng/mL
* = p<0.05 / ** = p<0.01 / *** = p<0.001 / **** = p<0.0001

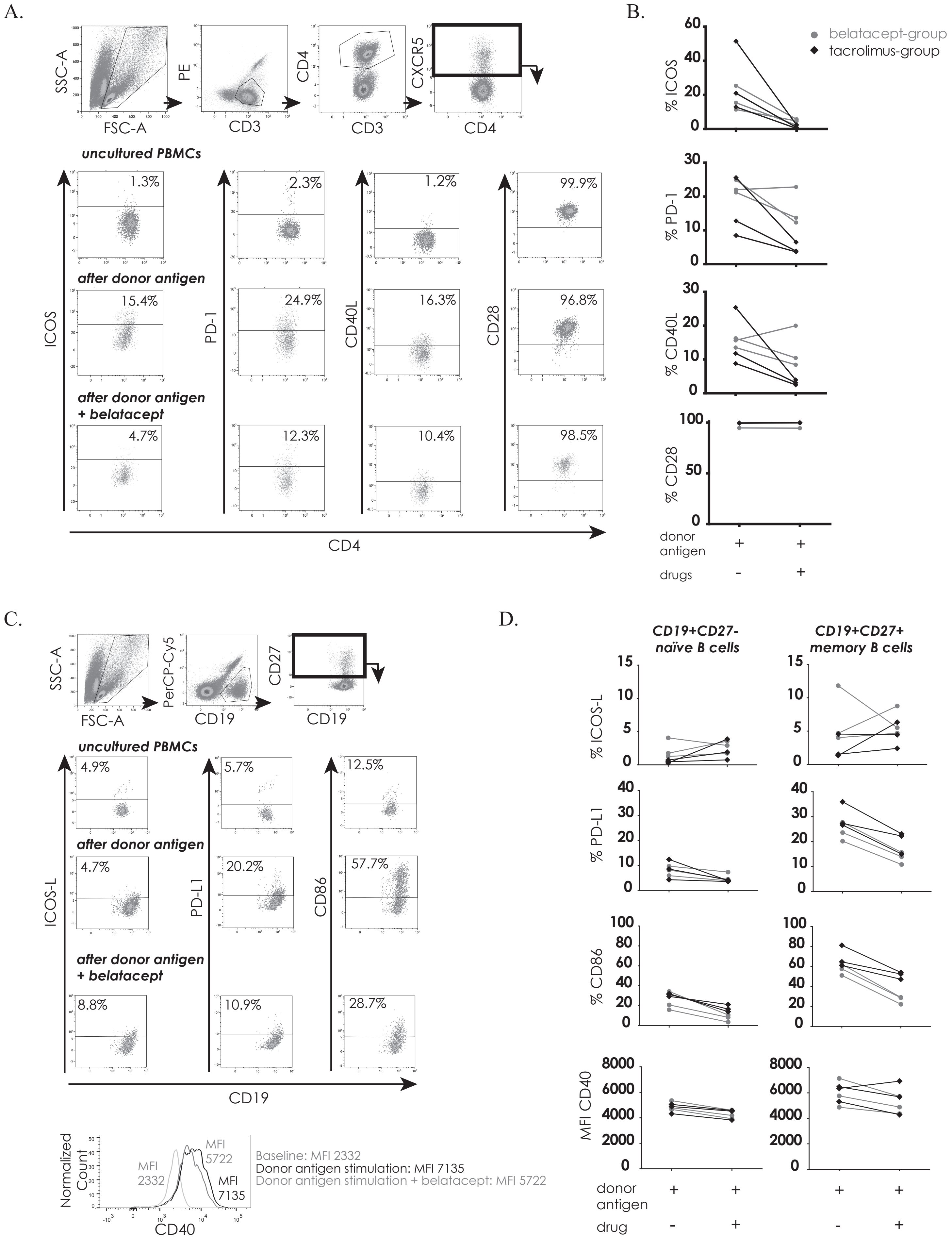

Supplementary Figure 4. Redundant co-stimulatory pathways in follicular T helper and B cells.
A typical example is shown for the expression of different co-stimulatory molecules on CD4+CXCR5+ T helper cells obtained from a belatacept-treated patient (A). PBMCs from tacrolimus-treated patients were gated the same way. The proportions of ICOS+, PD-1+, CD40L+ and CD28+ within CD4+CXCR5+ T helper cells are depicted for n=3 belatacept-treated patients (dots) and n=3 tacrolimus-treated patients (diamonds) in the presence or absence of belatacept 10 µg/mL or tacrolimus 10 ng/mL, respectively (B). A typical example is shown for the expression of different co-stimulatory molecules on memory (CD27+) B-cells obtained from a belatacept-treated patient 3 months after transplantation (C). Naïve (CD27-) B cells and B cells from tacrolimus-treated patients were gated the same way. The proportions of ICOS-L+, PD-L1+, and CD86+, and the Median Fluorescence Intensity (MFI) for CD40 within CD19+CD27- naïve and CD19+CD27+ memory B-cells are depicted for n=3 belatacept-treated patients (dots) and n=3 tacrolimus-treated patients (diamonds) in the presence or absence of belatacept 10 µg/mL or tacrolimus 10 ng/mL, respectively (D).

N.B.: All materials were obtained in stable, non-rejecting patients 3 months after transplantation. No statistical analyses were conducted.
