# Supplementary material for: Belatacept Does Not Inhibit Follicular T Cell-Dependent B-Cell Differentiation in Kidney Transplantation
Source: Front Immunol. 2017 May 31;8:641. doi: 10.3389/fimmu.2017.00641 (PMC5450507; doi:10.3389/fimmu.2017.00641)
Supplement: Supplementary file 2 [file table_1.docx]

**Supplementary Tables**

**Belatacept Does Not Inhibit Follicular T Cell-Dependent B-Cell Differentiation in Kidney Transplantation**

Gretchen N. de Graav^*^, Dennis A. Hesselink^*^, Marjolein Dieterich^*^, Rens Kraaijeveld^*^, W. Verschoor^*^, Dave L. Roelen^†^, Nicolle H.R. Litjens^*^, Anita S. Chong^‡^, Willem Weimar^*^, Carla C. Baan^*^

^*^Department of Internal Medicine, Section Transplantation and Nephrology, Erasmus MC, University Medical Center, Rotterdam, the Netherlands; ^†^Department of Immunohematology and Blood Transfusion, Leiden University Medical Center, Leiden, the Netherlands; ^‡^Department of Surgery, The University of Chicago, Chicago, Illinois, The United States of America

**Supplementary Table 1:** Inclusion and exclusion criteria

| *Inclusion criteria:* |
| --- |
| - Recipient of a first or second renal allograft |
| - Recipient of a living donor (related or unrelated) |
| - Current or historical panel-reactive antibodies (PRA) < 30% |
| - ≥1 HLA-DR mismatch |
| *Exclusion criteria:* |
| - Recipient <18 years of age at time of transplantation |
| - Recipient of a deceased donor |
| - Recipient of a third (or more) renal allograft |
| - Recipient of a non-renal organ transplant (present, past or near-future) |
| - Recipient of an AB0-incompatible allograft |
| - Recipient with a historically positive cytotoxicity-dependent cross-match |
| - Recipient with a history of lymphoma |
| - Recipient with a seronegative or unknown EBV status |
| - Recipient with HIV, hepatitis B or C, and/or untreated latent tuberculosis |
| - Recipient with a high risk for polyoma virus-associated nephropathy, which is mostly due to BK virus infection |
| - Recipient who already uses tacrolimus pre-transplantation |
| - Pregnancy |

**Supplementary Table 2:** Monoclonal antibodies

| **Experiment** | **Markers** | **Monoclonal antibodies** | **Firms** | **Added (µL)*** |
| --- | --- | --- | --- | --- |
| MLRs using PBMCs for Tfh-B-cell interaction (*n*=40 patients before and after transplantation) | CD3 | Brilliant Violet 510 | BioLegend, San Diego, CA | 0.25 |
|  | CD4 | Brilliant Violet 421 | BioLegend, San Diego, CA | 2 |
|  | CXCR5 | Alexa Fluor 647 | BD Pharmingen, San Diego, CA | 0.25 |
|  | PD-1 (CD279) | APC-Cy7 | BioLegend, San Diego, CA | 10 |
|  | CD8 | PerCP | BD Biosciences, Franklin Lakes, NJ | 5 |
|  | IL-21 | PE | eBioscience, San Diego, CA | 0.5 |
|  | CD19 | Brilliant Violet 510 | BioLegend, San Diego, CA | 5 |
|  | CD24 | APC | eBioscience, San Diego, CA | 5 |
|  | CD27 | PE-Cy7 | eBioscience, San Diego, CA | 1 |
|  | CD38 | Brilliant Violet 421 | BioLegend, San Diego, CA | 1 |
|  | IgD | APC-Cy7 | BioLegend, San Diego, CA | 5 |
|  | CD86 | PE | Beckman Coulter, Brea, CA | 10 |
|  | IL-10 | PE | BD Biosciences, Franklin Lakes, NJ | 0.5 |
|  | TNFα | PerCP-Cy5.5 | BD Biosciences, Franklin Lakes, NJ | 2 |
| MLRs using PBMCs for redundant costimulatory pathways (*n*=6 patients after transplantation) | CD3 | Brilliant Violet 510 | BioLegend, San Diego, CA | 0.25 |
|  | CD4 | Brilliant Violet 421 | BioLegend, San Diego, CA | 2 |
|  | CXCR5 | Alexa Fluor 647 | BD Pharmingen, San Diego, CA | 0.25 |
|  | PD-1 (CD279) | APC-Cy7 | BioLegend, San Diego, CA | 10 |
|  | CD40-Ligand (CD154) | FITC | BioLegend, San Diego, CA | 5 |
|  | ICOS (CD278) | PE-Cy7 | BioLegend, San Diego, CA | 0.5 |
|  | CD28 | PerCP-Cy5.5 | BD Biosciences, Franklin Lakes, NJ | 10 |
|  | CD19 | Brilliant Violet 510 | BioLegend, San Diego, CA | 5 |
|  | CD27 | PE-Cy7 | eBioscience, San Diego, CA | 1 |
|  | PD-Ligand 1 (CD274) | Brilliant Violet 421 | BioLegend, San Diego, CA | 2 |
|  | CD40 | FITC | BioLegend, San Diego, CA | 1 |
|  | ICOS-Ligand (CD275) | APC | BioLegend, San Diego, CA | 1 |
|  | CD86 | PE | Beckman Coulter, Brea, CA | 10 |
| MLRs in the presence of various concentrations of belatacept or tacrolimus (*n*=6 independent experiments) | CD19 | Brilliant Violet 510 | BioLegend, San Diego, CA | 5 |
|  | CD27 | PE-Cy7 | eBioscience, San Diego, CA | 1 |
|  | CD80 | APC | BioLegend, San Diego, CA | 5 |
|  | CD86 | PE | Beckman Coulter, Brea, CA | 10 |
| Co-cultures of isolated Tfh and memory B-cells (*n*=6 independent experiments) | CD4 | PerCP | BD Biosciences, Franklin Lakes, NJ | 10 |
|  | CXCR5 | Alexa Fluor 647 | BD Pharmingen, San Diego, CA | 0.25 |
|  | PD-1 (CD279) | APC-Cy7 | BioLegend, San Diego, CA | 10 |
|  | CD19 | Brilliant Violet 510 | BioLegend, San Diego, CA | 5 |
|  | CD27 | PE-Cy7 | eBioscience, San Diego, CA | 1 |
|  | CD38 | Brilliant Violet 421 | BioLegend, San Diego, CA | 1 |
|  | CD86 | PE | Beckman Coulter, Brea, CA | 10 |

***** The monoclonal antibodies were titrated to the optimal concentrations to discriminate between the positive and negative fraction for the tested marker. The depicted amounts of monoclonal antibodies were added to 100 µL cell suspension.

**Supplementary Table 3:** Characteristics of study subjects

| **Characteristics** | **Total randomized (*n*=40)** | **Randomized** | | **p** |
| --- | --- | --- | --- | --- |
|  |  | **belatacept (*n*=20)** | **tacrolimus (*n*=20)** |  |
| Age at transplantation (years) | 55 (21-76) | 57 (25-76) | 55 (21-76) | 0.88 |
| Gender (female) | 10 (25%) | 6 (30%) | 4 (20%) | 0.72 |
| HLA A mismatch (mean ± SD) | 1.2 (±0.6) | 1.1 (±0.7) | 1.4 (±0.5) | 0.13 |
| HLA B mismatch (mean ± SD) | 1.4 (±0.5) | 1.3 (±0.5) | 1.5 (±0.5) | 0.51 |
| HLA DR mismatch (mean ± SD) | 1.2 (±0.4) | 1.1 (±0.4) | 1.3 (±0.4) | 0.70 |
| HLA total mismatch (mean ± SD) | 4.0 (±1.1) | 3.5 (±1.1) | 4.1 (±1.1) | 0.07 |
| current PRA | 0% (0-17%) | 0% (0-5%) | 0% (0-17%) | 0.30 |
| highest PRA | 4% (0-21%) | 4% (0-6%) | 4% (0-21%) | 0.78 |
| CMV seropositivity at transplantation | 22 (55%) | 10 (50%) | 12 (60%) | 0.75 |

Data represent medians (plus ranges) for continuous variables and numbers (plus percentages) for categorical variables, unless otherwise specified. Two-sided p values comparing the two treatment arms result from the Mann-Whitney U test for comparing continuous variables or the Fisher’s exact tests for comparing categorical variables. Two-sided p<0.05 was considered statistically significant.

CMV, cytomegalovirus; HLA, human leukocyte antigen; PRA, panel reactive antibodies; SD, standard deviation.
